# Biosorption of arsenic (III) from aqueous solution using calcium alginate immobilized dead biomass of *Acinetobacter* sp. strain Sp2b

**DOI:** 10.1038/s41598-024-60329-7

**Published:** 2024-04-30

**Authors:** Renu Khandelwal, Sneha Keelka, Neha Jain, Prachi Jain, Mukesh Kumar Sharma, Pallavi Kaushik

**Affiliations:** 1 Centre for Advanced Studies, Department of Zoology, University of Rajasthan, Jaipur, Rajasthan 302004 India; 2 Department of Zoology, SPC Government College, Ajmer, Rajasthan 305001 India

**Keywords:** Arsenic, Biosorption, Biomass, Calcium alginate beads, Isotherms, Kinetics, Thermodynamics, Chemical biology, Microbiology

## Abstract

This study presents a novel biosorbent developed by immobilizing dead Sp2b bacterial biomass into calcium alginate (CASp2b) to efficiently remove arsenic (As^III^) from contaminated water. The bacterium Sp2b was isolated from arsenic-contaminated industrial soil of Punjab, a state in India. The strain was designated *Acinetobacter sp.* strain Sp2b as per the 16S rDNA sequencing, GenBank accession number -OP010048.The CASp2b was used for the biosorption studies after an initial screening for the biosorption capacity of Sp2b biomass with immobilized biomass in both live and dead states. The optimum biosorption conditions were examined in batch experimentations with contact time, pH, biomass, temperature, and As^III^ concentration variables. The maximum biosorption capacity (q_max_ = 20.1 ± 0.76 mg/g of CA Sp2b) was obtained at pH9, 35 ° C, 20 min contact time, and 120 rpm agitation speed. The isotherm, kinetic and thermodynamic modeling of the experimental data favored Freundlich isotherm (R^2^ = 0.941) and pseudo-2nd-order kinetics (R^2^ = 0.968) with endothermic nature (ΔH° = 27.42) and high randomness (ΔS° = 58.1).The scanning electron microscopy with energy dispersive X-ray (SEM–EDX) analysis indicated the As surface binding. The reusability study revealed the reasonable usage of beads up to 5 cycles. In conclusion, CASp2b is a promising, efficient, eco-friendly biosorbent for As^III^ removal from contaminated water.

## Introduction

Arsenic (As) is one of the most hazardous metalloids^1^ that has a high chance of reaching the general public via contaminated food and water, agricultural products, irrigated crops, industrial processes, and cigarette use. Chronic exposure above the permissible dose (WHO-10 μg/L) has several harmful consequences on human health, including lung and skin cancers, a variety of neurological, cardiovascular, and excretory problems, pigmentation of the skin and nails, and other fatal conditions^2,3^.

As induced toxicity primarily depends on its chemical form, with inorganic forms reported to incur more significant toxicological implications^2^. In nature, the most prevalent inorganic forms are the trivalent (As^III^) and pentavalent (As^V^) forms. Due to the higher reactivity with biological material, As^III^ causes higher toxicity than As^V^^4,5^.

Globally, over 100 countries have been reported with arsenic contamination in groundwater above the WHO permissible limit (0.01 ppm), with Asia and Europe being the worst affected^6^. The heavy exploitation of hard rock and alluvial aquifers to compensate for the lack of clean surface water brings elevated levels of As to the surface^6^. The surplus addition to the natural arsenic contamination occurs due to industrial and agricultural additive waste release^6^. Thus, there is an emergent need to reduce or remove high levels of arsenic from the potential exposure sources to prevent or reduce the harmful impact of As exposure. Various physicochemical and biological techniques, like oxidation, coagulation, ion exchange, membrane techniques, and adsorption, are available to reduce or remove As contamination^7–9^.

The chemical and membrane-based techniques are usually expensive, produce secondary waste, and thus are not considered eco-friendly. The main benefit of biological treatment over physicochemical treatment is that this does not burden the environment with secondary chemical pollutants. Therefore, natural materials are often preferred to develop eco-friendly and cost-effective bioremediation methods to deal with environmental contaminants like heavy metals. Bioremediation is an economical and sustainable approach, free from other limitations, being practiced widely under several As-contaminated environments^10,11^. The well-known bioremediation strategies to combat metal pollution include bioaccumulation, oxidation, reduction, precipitation, coagulation, and biosorption^12–17^.

Biosorption is the best technique for developing arsenic remediation, involving the biological surface properties and chemical or physical interaction with the pollutant. The natural material that can adsorb the metal pollutant is called the sorbent or biosorbent, and the contaminant is called the sorbate or biosorbate. This technique provides the best alternative for removing toxic metals from polluted streams by live or dead natural materials^14,18^.

Numerous reports on arsenic removal using biological materials like plants or microbes like bacteria, fungi, algae, and yeast in living or dead states are available in the literature^14,19–25^. The diversified bacterial population from contaminated or uncontaminated sources have profound potential for the development of biosorbent with high adaptability and tolerance, ease of culturing and diverse surface properties make them a valuable bi- resource^23,26,27^. As biosorption does not involve bacterial metabolism, the inactive or dead biomass is preferred choice of workers to avoid the use of growth media and stringent growth conditions^28,29^.

Such biomass can also be easily immobilized on suitable substrate to enhance the efficiency and applicability of biosorption process with easy handling, separation and reusability^30,31^. The potential for regenerating adsorbents and the user-friendly applicability at household and rural levels make adsorption with immobilization an attractive choice. Various immobilizing agents or biomass carrier are known like silica gel, zeonite, diatomaceous earth and synthetic or natural polymer matrixes like polyacrylamide, polyvinyl alcohol, cellulose, agar, carageenan, chitosan, alginate etc^30^. The simple gelation and great biocompatibility, enhanced mechanical strength and resistance to environmental stress of alginate with CaCl_2_ crosslinking is preferable bacterial biomass immobilizing agent with evidences to improve biosorption^31–33^. The application-based study to standardize the biosorption potential of any such system requires the efficacy analysis in various environmental variables^34,35^. The nature of such biosorption can be estimated by biosorption isotherms, kinetics, and thermodynamics, obtained after the computation of experimental results of concentration, time, and temperature variables, respectively^36–38^.

The quest to explore and standardize newer bacteria based biosorbent with the efficiency of biosorption at high arsenic contamination levels with wider applicability has led to the development of the framework of the present study. The specific aim of the study was development and optimization of As^III^ biosorbent using As^III^ tolerant bacteria.

Thus, the As^III^ tolerant bacteria Sp2b was isolated from an arsenic-contaminated site in Punjab, India. This bacteria was characterized for minimum inhibitory concentration (MIC) for As^III^, surface functional properties by Fourier transform infrared spectroscopy (FTIR), and surface morphological study by scanning electron microscopy (SEM).The identification of the bacterium was done as *Acinetobacter sp.* Sp2b by sequencing and aligning the 16S rDNA sequence with the National Centre for Biotechnology Information (NCBI) database, and analysis of biochemical properties. Prior studies on *Acinetobacter* provide information about its tolerance to metals and antibiotics and its essential role in metal removal^39–42^. Although, various biosorbent for removal of As^III^ have been reported in literature^43^. But the modeling studies for As^III^ removal using immobilized *Acinetobacter* sp.Sp2b is not known.

The dead biomass of Sp2b was selected for optimization and modeling on the basis of efficacy and applicability in initial screening using live and dead biomass with and without immobilization.The biosorption experiments were conducted with condition variables of pH, temperature, initial As^III^ dose and biomass to determine the optimum conditions and isotherm, kinetics and thermodynamics modeling. We have also suggested the possible mechanism of biosorption based on the surface binding analysis using SEM–EDX and mapping.

## Materials and methods

### Selection of the study area

Soil samples were collected from the industrial area of Ludhiana district (30.5312.60″ N, 75° 54′ 17.57″ E) Punjab, a state of India, for isolation and characterization of arsenic-tolerant bacteria.

### Isolation of Arsenic tolerant bacteria

The soil was serially diluted and plated on nutrient agar(procured from HiMedia Laboratories Private Limited, India) plates containing 1 g/L of As^III^ (sodium-meta-arsenite, NaAsO_2_, procured from HiMedia Laboratories Private Limited, India). The arsenic tolerant bacterial strain was obtained as pure colonies on separate nutrient agar (NA) plates by repeated quadrant streaking.

### Determination of minimum inhibitory concentration (MIC)

The tolerance to As^III^ was estimated by exposing the bacterial isolate to variable doses of Sodium-meta-arsenite(1–8 g/L) with calculated As^III^ concentration in the range of 13.35–61.61 mM)in nutrient broth(pH-7.4 ± 0.3) at 35 °C with shaking at 120 rpm (Orbital Shaking Incubator, REMI). The growth at each dose was determined as optical density (600 nm) at 24 h, 48 h, and 72 h incubation period using a UV–visible spectrophotometer (Make-Systronics; Type-106). The lowest dose of arsenic, which showed complete growth inhibition, was considered the MIC^44–46^.

### Identification and characterization of strain Sp2b

The genomic DNA was extracted from pure Sp2b culture, and 16S ribosomal DNA sequence was amplified using 16S rDNA universal primers. The sequencing of the PCR product was done at Dextrose Technologies Private Limited, Bangalore, India.

#### Genomic DNA extraction

The fresh culture of Sp2b was homogenized with extraction buffer (1 mL) and mixed with an equal volume of phenol: chloroform: isoamyl alcohol (25:24:1) followed by centrifugal separation of upper aqueous phase (14,000 rpm, 15 min). To the upper aqueous phase, an equal volume of chloroform: isoamyl alcohol (24:1) was added and mixed. This tube was centrifuged (14,000 *rpm*) at room temperature (10 min). The DNA present in the upper aqueous phase was precipitated by adding 0.1 volume of 3 M sodium acetate (pH 7.0) and 0.7 volume of isopropanol and incubated at room temperature (15 min) followed by centrifugal separation of DNA pellet (14,000 rpm for 15 min at 4 °C). The obtained DNA pellet was washed with 70% ethanol (twice), followed by a very brief washing with 100% ethanol and air drying. The washed DNA pellet was then dissolved in TE buffer (Tris–Cl 10 mM pH 8.0, EDTA 1 mM). To avoid any impurity of RNA, 5 µl of DNase-free RNase A (10 mg/mL) was added to the DNA to remove RNA.

#### PCR amplification of 16S gene

The obtained DNA was used for PCR amplification with 10pM of each 16S forward (F243; GGATGAGCCCGCGGCCTA) and 16S reverse (R1378; CGGTGTGTACAAGGCCCGGGAACG) universal primers using Uni-directional high–fidelity PCR polymerase^47,48^. The PCR reaction was performed using the Taq Master Mix. Each PCR amplification well contained 1 μL of DNA, 2 μL each of 16s Forward and 16s Reverse Primer, 4 μL of dNTPs (2.5mMeach), 10 μL of 10X Taq DNA polymerase Assay Buffer, 1 μL of Taq DNA polymerase enzyme (3U/mL) and 30 μL of water to make the 50 μL total volume of reaction mixture. The PCR was run with an initial denaturation at 94 °C for 3 min and further run for 30 cycles with denaturation at 94 °C for 1min, annealing at 50 °C for 1min, extension at 72 °C for 2 min, and elongation at 72 °C for 7min.

#### 16SrDNA sequencing

The PCR product of size ~ 1.5 kb was isolated and sequenced uni-directionally using ABI 3130 Genetic Analyzer (sequencing machine), big dye terminator version 3.1 (cycle sequencing kit), BDTv3-KB-Denovo_v 5.2 (Analysis protocol), using Seq Scape v 5.2 (data analysis software).The sequencing mixture was composed of 4μL of big dye terminator ready reaction Mix, 1 μL of DNA Template (100 ng/mL), 2μL of Primer (10 pmol/λ), and 3μL of Milli Q Water to make 10 μL sequencing reaction. The PCR conditions included 25 cycles with an initial denaturation at 96 °C for 5 min followed by 25 cycles of denaturation (96 °C for 30 s), hybridization (50 °C for 30 s), and elongation (60 °C for 1.30 min).

#### Sequence alignment and phylogenetic tree

The16S rDNA genes sequence data of the bacterial isolate was aligned and analyzed to identify the bacteria and its closest neighbors through BLASTn (nucleotide BLAST, database 16S rRNA sequences) of National Centre for Biotechnology Information (http://www.ncbi.nlm.nih.gov/blast). Based on the maximum identity score, the first 10 sequences were selected, and the phylogenetic tree was constructed based on the neighbor-joining (NJ) tree method. The tree was generated using the MEGA 11 software package to evaluate the tree topology and calculate the statistical significance of branch points; bootstrapping was performed for the tree with 1000 replicates. The 16S rDNA sequence was deposited in the GenBank to generate the accession number.

#### Characterization of strain Sp2b

*Colony morphology, Staining, and Biochemical tests:-*To support the results of bacterial identification by 16S rDNA sequencing, specific characteristics of Sp2b were studied as per *Bergey’s Manual of Determinative Bacteriology *(1994)^49^.

##### SEM (scanning electron microscopy)

The surface morphology of strain Sp2b was studied using SEM. For SEM analysis, the lyophilized bacterial cells were placed on a carbon tape, coated with gold, and inserted in a Field Emission Scanning Electron Microscopy (FESEM-JEOL India Pvt. Ltd, Model number-JSM7610FPLUS) chamber and examined at Manipal University, Jaipur, Rajasthan, India.

##### FTIR (fourier transform infrared)

The FTIR spectra of surface functional groups of the bacterial isolate were analyzed in the following conditions: Kbr method^50^, infrared spectral range of 400–4000 cm^–1^, and resolution of 4 cm^–1^.

### Biosorption studies

The biosorption studies using the bacterial isolate were conducted in two parts. In the first part, we estimated the biosorption capacity of Sp2b biomass in live and dead states before and after immobilization at variable time intervals. This initial study was designed to evaluate the effectiveness and applicability of Sp2b biomass after immobilization for arsenic removal. In the next part, the biosorption potential of calcium alginate immobilized dead bacterial biomass beads (CASp2b) was analyzed under condition variables of pH, temperature, initial As^III^ concentration, and biomass dose. We have further computed the experimental results for Langmuir and Freundlich isotherms, pseudo-1st-order and pseudo-2nd-order kinetics, and thermodynamics modeling. The surface morphology and elemental composition of the As^III^ exposed and unexposed calcium alginate immobilized dead bacterial biomass beads were also analyzed using SEM, SEM–EDX, and mapping.

The biosorption potential of the sorbents was expressed in terms of removal percentage and biosorption capacity as per Eqs. (1) and (2)^51–54^.

1 $$\mathrm{As\,\, \% \,\, removal }=\frac{Ci-Cf}{Ci}\times 100,$$

where *ci* and *cf* refer to the initial and final concentration of As^III^ (AAS estimation).

Equation (2) computes the quantity of arsenic sorbed by the immobilized biomass beads at equilibrium.

2 $$\mathrm{Arsenic \,\,biosorption \,\,capacity }\left({\text{qe}}\right)=\frac{\left(Ci-Ce\right)V}{M},$$

where *ci* and *ce* refer to the initial concentration and concentration of arsenic (mg) at equilibrium, as per the AAS estimation, V = volume of arsenic-containing deionized water (L), M = wet weight of biomass beads (g).

#### Bacterial biomass preparation and immobilization

The bacterial isolate was cultured under optimum growth conditions for 48 h, and biomass was separated into live and dead states. The dead biomass was obtained by autoclaving at 121 °C for 15 min at 15lbs pressure. The biomass separation and preparation were done by thrice sequential centrifugal separation (5000 rpm for 15 min) and washing in sterile deionized water. To prepare the calcium alginate immobilized beads biomass beads, 1% (wt/v) sodium alginate powder and 1% wet weight/volume (wt/v) of bacterial biomass were mixed in DDW (double distilled water) using a glass rod and magnetic stirrer. The spherical beads were prepared by drop extrusion of this viscous solution using a 10 mL syringe with a 1.0 mm needle size with an average speed of 1 drop in 2 s into CaCl_2_ (2%, w/v) solution under regular stirring. These beads were left in the solution for about 2 h for curing, followed by multiple times washing to remove excess unbound Ca^2+^ to obtain 2 to 3 mm immobilized calcium alginate Sp2b beads (CASp2b) (Fig. 3a). The only calcium alginate beads were also prepared using a similar method without the biomass. The biomass dose used in all biosorption experiments was 2 g in 100 mL (2%) of As^III^ amended deionized water except the biomass variation experiment as per literature study^55^.

#### Preliminary biosorption study using live and dead bacterial biomass with or without immobilization

The preliminary biosorption study was conducted using 2% (2 g/100 ml) of all the biomass variables, i.e. Sp2b live and dead biomass, and immobilized live and dead biomass in aqueous solution containing As^III^ (200 mg/L)^56^ at pH 9.35 °C for variable time intervals (5–120 min). The equilibrium time and maximum biosorption capacity (q_max_) comparison of each biomass (Fig. 3a) indicated the highest biosorption efficiency of CASp2b. Thus, CASp2b was selected for biosorption condition optimization and isotherm, kinetics, and thermodynamics modeling. The time after which no significant increase in biosorption capacity is observed is considered the equilibrium time^57,58^.

#### Biosorption experimentation setup using CASp2b

##### Effect of variables (As^III^)concentration, pH, temperature, biomass

The biosorption experimentations were conducted using the variability of one component (Initial As^III^ concentration, pH, temperature, and biomass) at a time to determine the optimum biosorption conditions. The isotherm modeling was conducted with the initial As^III^ concentration variables (100, 200, 500, 1000 and 1500 mg/L) biosorption results. Meanwhile, the biosorption kinetics was determined by time variable (5–120 min) results. The results of temperature variability (20 °C, 35 °C, 50 °C) were used to determine the thermodynamic nature of the biosorption process. The biomass dose variation (1%, 2%, 4%) provided an estimate of suitable biomass dose for biosorption.

##### The experiment of reusability

The reusability of the CASp2b beads was examined in seven cycles of sorption and desorption. The sorption experiments were conducted at optimum conditions (pH 9, 35 °C, 1000 mg/L of As^III^, 20 min contact time). In contrast, the desorption of bound arsenic on beads was done by immersing the beads in 50 ml of 0.1 M HNO_3_ for 3 h and shaking them at 100 rpm. After each sorption–desorption cycle, the beads were neutralized in 2% CaCl_2_ and washed in distilled water several times to remove unbound calcium^55^.

##### SEM–EDX and mapping of CASp2b

For the SEM imaging, the lyophilized biomass beads of Sp2b were placed on a carbon tape, coated with gold, and inserted in a Field Emission SEM chamber. Energy dispersive X-ray spectroscopy (EDX) was also applied for the surface elemental composition along with layered mapping of different components of the biosorbent surface before and after As exposure(FESEM-JEOL India Pvt. Ltd, Model number-JSM7610FPLUS, at Manipal University, Jaipur).

#### Methods of arsenic estimation

The biosorption potential of the biosorbent was studied by quantitative estimation of arsenic in the biosorbent exposed to As^III^ amended deionized water after suitable contact time. Arsenic concentration was determined using the modified molybdenum blue method^12^^,^^59^ and atomic absorption spectrophotometry^60^.

##### Arsenic doses

The As^III^ dosing for the experimental setup was prepared from the appropriate dilution of As^III^ stock solution. The As^III^ stock solution was prepared using sodium meta-arsenite (NaAsO_2_) procured from HiMedia Laboratories Private Limited,India. The As^III^ doses mentioned in the figures represent the dose of NaAsO_2_ used in the experiments (Figs. 4, 5 and 6). However, the estimated initial *ci* and final *cf* As^III^ concentrations, which represent the actual As^III^ concentration, have been used for all the calculation purposes.

##### Modified molybdenum blue method

Estimation of Arsenic concentration was done after oxidation of As^III^ to As^V^(arsenate) by using hydrogen peroxide (H_2_O_2_), which is a strong oxidant^61^, followed by estimation of total As^V^ by modified molybdenum blue method. This method estimates the As^V^ concentration in terms of the blue-colored complex of molybdo-arsenate, which is read using a UV–vis spectrophotometer after determining the absorption peak at 840 nm.The As^III^ containing water samples were centrifuged and filtered to remove biomass, and 0.3 mL of the resulting supernatant was mixed with H_2_O_2_ (0.2 mL), 50% H_2_SO_4_ (0.4 mL), 3% Na_3_MoO_4_ (0.4 mL), 2% freshly prepared ascorbic acid (0.2 mL) and then heated at 90 °C in the water bath for 20 min. The samples were then cooled to ambient temperature, and ultrapure water was added to make the total volume of 10 mL in the volumetric flask. A standard absorbance curve with different concentrations of sodium-meta-arsenite was prepared to convert the A_840_ into arsenic concentrations^12,59^.

##### Atomic absorption spectrophotometric method for as estimation

The As estimation was done in soil samples and As^III^ containing deionized water before and after the biosorption experimentations (after biomass separation) using furnace Atomic Absorption Spectrophotometer (AAS, model A Analyst 100, Perkin Elmer) with standard methodology^60^ at Team test house, Jaipur.

#### Safety and disposal

The treated biomass containing As^III^ were collected safely and put in yellow colored non-chlorinated plastic bags which were collected, transported, treated and disposed off as per under Biomedical Waste Management (BMWM) Rules, 2016 within 48 h. All these bags were labelled with tag of time, date, type quantity and symbol of Bio Hazard. The waste is pretreated by autoclaving or chemical disinfectants followed by incineration in two chambered incinerator with retention time of two seconds in secondary combustion chamber with suitable devices for controlling air pollution to follow with updated emission standards under BMWM Rules, 2016^62^.

##### Statistical analysis

All the experiments were performed in triplicates, and the observations were expressed in terms of mean ± standard error.

## Results and discussion

### Study area

The soil of the industrial area of Ludhiana district in Punjab,a state of India, was found to contain 7.18 mg arsenic per kg of soil as per the soil AAS analysis in the present study. Many regions in Punjab suffer from heavy metal pollution^63–65^. The arsenic contamination is beyond safe limits in more than 13 districts of Punjab. Most arsenic-contaminated areas fall in the Majha Belt of Punjab, including Gurdaspur and Tarn Taran, Amritsar, near the Ludhiana districts^66^. As per reports, the Arsenic source in the study area’s soil and groundwater (Punjab)is due to geogenic and anthropogenic sources^67^. Natural contamination appears in the groundwater, with 99 samples of groundwater from industrial regions of Punjab found contaminated with arsenic and other environmentally sensitive elements (ESEs)^68^. The anthropogenic activities are responsible for additional metal burdens in the soil and water system due to the indiscriminate disposal of industrial waste, as the area around industries shows elevated contamination levels^68,69^.

### Isolation of As^III^ tolerant bacterium

The bacterial strain Sp2b was obtained as pure colony on As^III^ containing NA plates. A similar method of isolation of arsenic-tolerant bacteria was adopted earlier, where the isolation source was found to be contaminated with arsenic from soil-receiving textile waste^45^, soil and water-receiving tannery waste^70^, and from arsenic-accumulating plant *Pteris vittata* growing near the lead–Zinc mine region^71^.

### Minimum inhibitory concentration (MIC)

**Figure 1:**
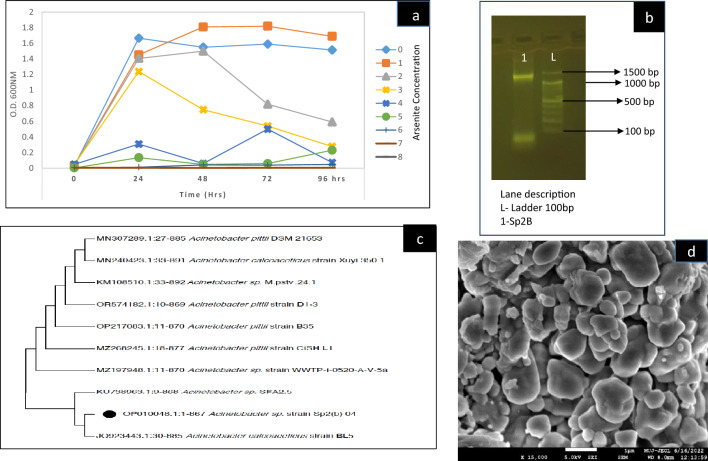
Identification of bacterium Sp2b (**a**) MIC with increasing doses of As^III^ (NaAsO_2_; 1–8 g/L) in Nutrient broth at 35 °C at 120 rpm with determination of MIC at 7 g/L; (**b**) Agarose gel electrophoresis image -First Lane (1) is of 16 S rDNA PCR product and the second lane (L) consists of DNA 100 bp ladder (100 bp-1500 bp)(original gel image is presented in Supplementary Fig. 1); (**c**) Phylogenetic tree was constructed based on neighbor-joining (NJ) tree method using MEGA 11 software; (**d**) SEM image at 15000X.

The bacterial isolate exhibited tolerance in response to increasing doses of As^III^ from 1 to 8 g/L (13.35–61.61 mM of As^III^) (Fig. 1a). The growth was higher at the dose of 1 g/L compared to control (0 g/L) from 48 to 96 h. At the next dose of 2 g/L, the growth of the bacterium was comparable to control (0 g/L) at 48 h of incubation. But unlikely to the control, there was a rapid decline in optical density in subsequent intervals (72 h and 96 h).The toxic effects of growth inhibition were observed in the subsequent doses (3–8 g/L) with complete growth inhibition at 7 g/L (53.88 mM of As^III^). Thus, the minimum inhibitory concentration (MIC) of As^III^ was determined with complete growth inhibition at 7 g/L for bacterial isolate Sp2b.Various ranges of MIC for As^III^ in bacteria are available in the literature. In our earlier report, four arsenite hypertolerant bacteria were isolated with MIC ranging between 23.9 and 62.2 mM^45^. In another study on arsenite and arsenate-tolerant bacteria,42 arsenite-resistant bacteria were isolated with MIC ranging from 5 to 40 mM^70^. High MIC (45 mM) was reported in the 116 arsenic tolerant bacteria isolated from roots of the arsenic hyper accumulator plant *P.vittata*^71^. Bacterial tolerance to arsenic is often associated with the presence of arsB and ACR3 arsenic transporter genes^71,72^. In these studies, the presence of these genes was confirmed with their PCR amplification using specific degenerate primers. The cited literature^71^ reported that 70.7% of 41 arsenic-resistant bacteria contained genes related to arsB and ACR3 families. It is also suggested that these genes be transferred horizontally in the bacteria residing in arsenic-contaminated regions.

### Identification and characterization of strain Sp2b

The genomic DNA extracted from the pure culture of Sp2b was checked qualitatively by agarose gel electrophoresis. The extracted DNA was used for 16S rDNA amplification, which yielded a single amplicon of approximately 1.5 Kb (Fig. 1b; Supplementary Fig. 1). The sequencing of the PCR product and alignment with 16S rDNA sequences in the NCBI database provided a similarity table from the top ten matches (Supplementary Table 1).

Based on the maximum identity score, the first 10 sequences were selected (Supplementary Table 1) and the phylogenetic tree was constructed based on the neighbor-joining (NJ) tree method. The closest matches with the 16S rDNA sequence of the bacterial isolate Sp2b were *Acinetobacter calcoaceticus* strain BL5 16S with accession number JQ923443 and (99.65% similarity), *Acinetobacter* sp. strain WWTP-I-0520-A-V-5a with accession number MZ197948 (99.54% similarity). Based on these results, the bacterial isolate Sp2b was taxonomically designated as *Acinetobacter* sp. strain Sp2b with OP010048 accession number obtained from GenBank. The phylogenetic tree (Fig. 1c) shows the systematic position of the isolate.

The bacteria of the genus *Acinetobacter* have received significant attention from the scientific community with its presence and tolerance to unfavorable conditions (heavy metal and antibiotic stress). *Acinetobacter calcoaceticus* (strain STP 14) has been reported to tolerate heavy metal stress like mercury, cobalt, copper, nickel, lead, and cadmium, along with co-resistance to tested antibiotics. This tolerance can be attributed to the differential expression of genes responsible for such resistance, evidenced by the difference in the protein bands of metal-exposed and unexposed bacteria^42^. These resistance genes can be genomic or plasmid-borne. The sequencing of plasmidome from an arsenic-tolerant *Acinetobacter lwoffii* (strainZS207) revealed the presence of 9 plasmids, out of which one megaplasmid (size-186.6 kb) was found to contain the heavy metal as well as arsenic resistance genes (*ars* genes)^73^.

In another study, arsenic tolerance has been reported in five species of *Acinetobacter (A. soli, A.venetianus, A.junii, A. baumannii, and A.calcoaceticus)*. The a*rsB* and *aoxB* genes were detected in the genomic and plasmid DNA by PCR amplification using specific primers. These isolates also accumulated arsenic as a function of time and concentration^39^*.* *Acinetobacter* sp. XS21 can oxidize arsenite and remove 70% of arsenic from the soluble-exchangeable fraction compared to the control^41^. *Acinetobacter* sp. FM4 is also a good biosorbent for other heavy metals(Cr^VI^, Cu^II^, and Ni^II^)^40^.

#### Colony morphology, staining, and biochemical tests

The bacterial isolate Sp2b formed cream-colored circular translucent colonies with lobate margins and no motility when grown at 37 °C for 48 h. The bacterium Sp2b was found to be acid-fast-negative and gram-negative cocco-bacilli. The bacterium was found negative for starch utilization, dextrose fermentation, arabinose fermentation, lactose fermentation, xylose fermentation, nitrate reduction, oxidase, and reductase activity. It was found positive for citrate utilization and indole production. These reports are in agreement with earlier studies on *Acinetobacter*^74^^,^^75^.

#### Scanning electron microscopy (SEM)

**Figure 2:**
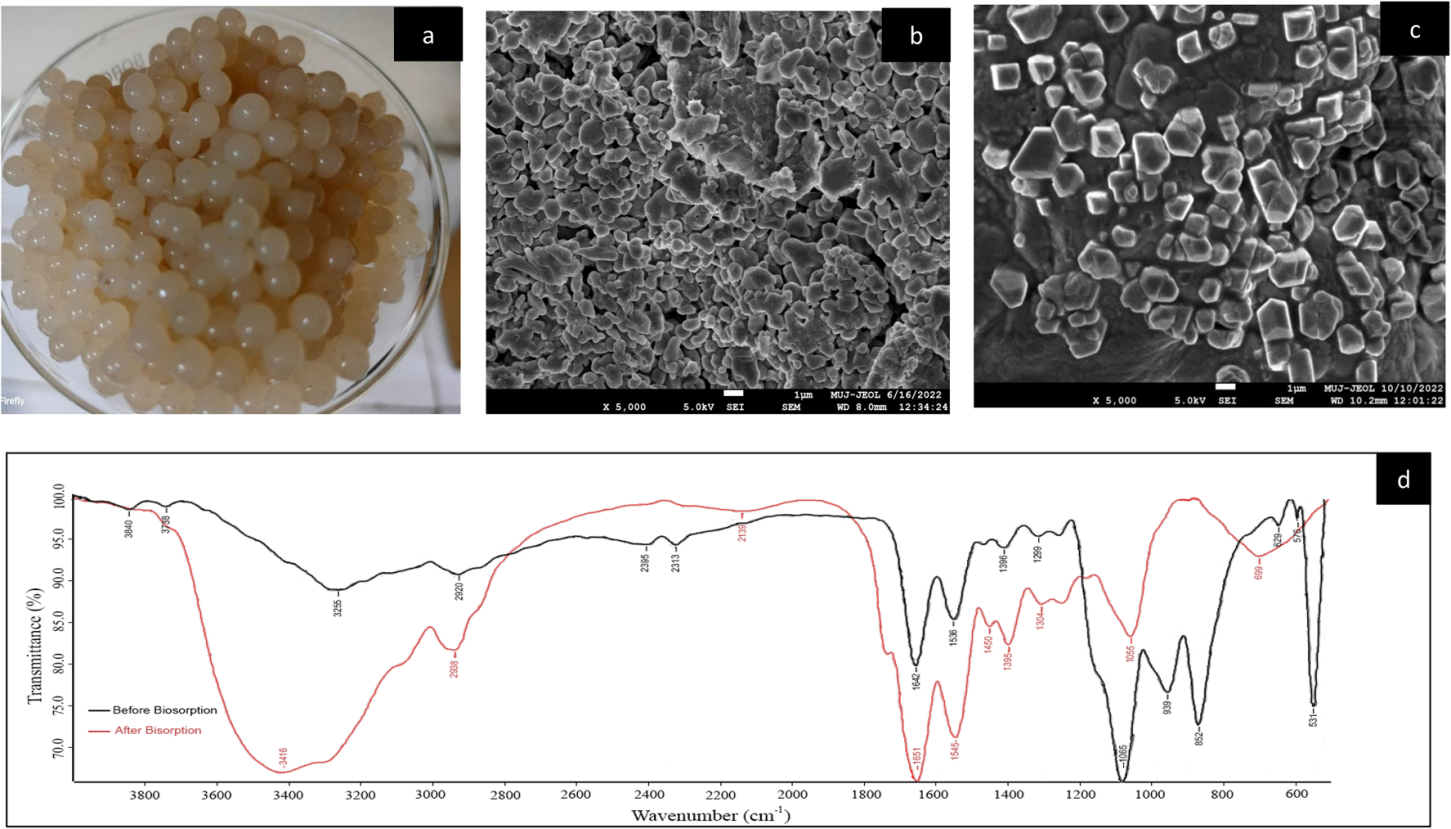
(**a**) Calcium alginate(CA) beads with biomass of Sp2b; (**b**) SEM images of CASp2b bacterial beads at 5000X; (**c**) SEM image As^III^ exposed CASp2b beads at 5000X; (**d**) FTIR analysis of Sp2b biomass before and after As^III^ treatment with characteristic wavelength peaks.

The outer morphology of strain Sp2b was studied using SEM images (Fig. 1d) as a large cocco-bacilli structure. Immobilization of Sp2b bacterial isolate biomass (Fig. 2a) was done to enhance the interaction between the sorbent and sorbate, which can significantly improve overall As^III^ biosorption. Before exposure to As^III^, SEM images of CASp2b appeared as interspaced rough, heterogeneous, mesh-type structure, which helps to increase surface area and provides more sites for adsorption, which favors heterogeneous surface adsorption (Fig. 2b). In a study on the selective removal of copper from a multi-metal mixture using calcium alginate beads, the SEM images showed a similar mesh-type structure to provide more sites for biosorption^55^. Other reports also observed a similar heterogeneous surface of calcium alginate beads^76^. After the exposure of CASp2b beads to As^III^, the surface morphology seems to differ with the presence of shiny, clearly visible crystalline patches in SEM images (Fig. 2c). In another study, the surface SEM images of calcium alginate beads prepared with crude extract of arsenate reductase enzyme entrapped in glutaraldehyde were highly porous. Still, after the exposure to arsenate, the surface profile was smooth and unwrinkled. These beads showed optimum As^V^ biosorptive potential^77^.

#### FTIR (fourier transform infrared)

FTIR analysis provided the information about the diverse surface functional groups of As^III^ treated and untreated Sp2b biomass in form of spectral peaks. The spectral analysis was done in the range of 400–4000 cm^−1^ with a resolution of 4 cm^−1^, following the methodology reported by Refs.^78,79^. The spectral ranges indicating specific functional groups on the surface of untreated biomass (Fig. 2d) included 2850–3300 cm^–1^ for Methyl group (C–H),1680–1750 cm^–1^ for carbonyl group(C=O), 1000–1300 cm^–1^ for C–O* (functional group), 3239–3550 cm^–1^ for alcohol (O–H) and broad distribution of 2500–3300 for acids (O–H). The FTIR results indicated the presence of methyl, carbonyl, carboxylic, and alcoholic functional groups on Sp2b surface which could potentially contribute to the binding of heavy metals^80^.

The FTIR results of the As^III^ treated biomass (Fig. 2d) revealed a noteworthy shift in the spectral band positions which indicates the active involvement and chemical modification of the surface functional groups during the As^III^ biosorption. The primary significant shift observed in the peaks at 3416 cm^–1^ corresponds to the O–H stretching band of alcohols or phenols. Additionally, peaks at 2938 cm^–1^ and 2139 cm^–1^ are attributed to C–H and C=C bonding of alkane, respectively. The peak at 1545 cm^–1^ signifies the N=O stretching band of a Nitro compound, while the peak at 1450 cm^–1^ represents the C=H stretching of alkane. The peak at 1055 cm^–1^ corresponds to the C–O group banding of aliphatic ether, and the functional group peak at 699 cm^–1^ is responsible for the C=C bonding of alkane. These identified As^III^ biosorption peaks and patterns are consistent with previous studies and highlight the involvement of various aliphatic, aromatic, and nitro compounds with hydroxyl amino groups in As^III^ biosorption^38^.

Furthermore, the sharpening of the band around 3200–3400 cm^−1^ confirms the predominant involvement of –OH groups in As^III^ biosorption^81^. According to Ref.^82^ carboxyl groups, are the main functional groups that undergo modification, creating favorable sorption sites for anionic species like As^III^. These negatively charged functional groups are crucial in forming crosslinking bonds with calcium alginate beads and arsenic. The presence of characteristic peaks in the FTIR spectra indicates the successful biosorption of As^III^ by CASp2b beads. The chemical adsorption between the functional groups of CASp2b and As^III^ is evident from the FTIR peak results, as discussed by Ref.^83^.

### Biosorption studies

#### Preliminary biosorption study using live and dead bacterial biomass with and without immobilization with contact time variability

**Figure 3:**
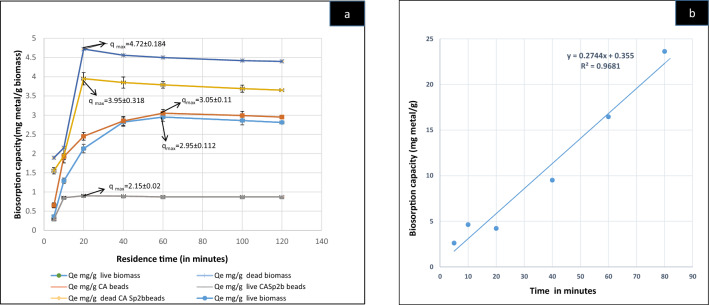
(**a**)Biosorption studies-Effect of time variation on biosorption capacity of un-immobilized and Calcium alginate immobilized Sp2b biomass in the dead and live state (at pH-9; As^III^ con.-200 mg/L; 35 °C;biomass-2% wt/v; 120 rpm) (**b**) Pseudo-2nd -order kinetics graph of CASp2b beads.

The results of this preliminary study indicated higher biosorptive potential of immobilized beads with comparably better biosorption in dead biomass immobilized beads (Fig. 3a). The comparison was made for biosorption capacity and equilibrium time attainment between all types of biomass. The equilibrium time for live and dead biomass was about 60 min and calcium alginate immobilized biomass was 20 min. Effective metal sorbents typically exhibit a characteristic pattern of initial rapid metal removal, followed by a gradual decline until equilibrium is reached, signifying no further increase in biosorption^67^. A parallel trend in metal biosorption has been observed with sorbents derived from activated teff straw^68^. In the case of algae biomass *Ulothrix cylindricum*^58^, reported an equilibrium time of 60 min. In contrast^81^ and ^82^ found that immobilized beads achieved equilibrium in As^III^ biosorption within 30 min. This initial rapid increase in biosorption capacity is attributed to the abundance of available adsorption sites. However, beyond a certain point, there is a saturation of sorption sites, leading to the attainment of equilibrium***.***

Metal absorption by non-living cells is viewed as a passive process. The utilization of dead biomass in passive biosorption leads to rapid uptake, achieving adsorption equilibrium within 30–40 min, as indicated by literature sources^84,85^. The mechanism of metal removal in live or dead biomass is different. In live state the active uptake of metal and further enzymatic modification or accumulation is involved^86^. In dead state the bacterial metabolism is abolished and the biosorption merely occurs due to the interaction of toxicant with bacterial surface functional groups^28,29^. Moreover, the inactivation by autoclaving is also found to improve the biosorption efficiency, this might be attributed due to the breakdown of bacterial surface structure to expose more functional groups for metal biosorption^87^. The wider use and applicability of dead biomass have been known to increase by immobilization on suitable substrate with advantage of ease of handling and separation ,larger surface area and improvement on biosorptive potential by many workers^30,31,33^. Due to the highest biosorption capacity observed with CASp2b (dead biomass immobilized beads) it was chosen for subsequent biosorption experiments.

##### Biosorption kinetics

The data of contact time variation were plotted using the pseudo-1st-order and pseudo-2nd-order models for the kinetics study. These models assume that the biosorption rate shall be proportional to the number of free active surface binding sites on the biosorbent in the proper power (1st or 2nd) for pseudo-1st -order or pseudo 2nd–order^29^.

##### Pseudo-1st-order kinetics

The equation proposed by Lagergren assumes that the change in absorption rate is proportional to the variation between the amount adsorbed and saturation concentration. The experimental data were plotted in the linear version of pseudo-1st-order kinetics with *ln(ci/ct)* on the Y axis and time (*t)* to determine the rate constant (K_1_) and R^2^ by the slope of experimental data using the Eq. (3)^88^ (Supplementary Fig. 2).

3 $$\frac{dqt}{dt}={K}_{1}\left({q}_{e}-{q}_{t}\right).$$

Pseudo -2nd-order kinetics-The Pseudo 2nd -order linear equation^56,88^ is written as,

4 $$\frac{t}{qt}=\frac{1}{K2{qe}^{2}}+\frac{t}{qe},$$

where *qe* and *qt* represent equilibrium sorption capacity and sorption capacity (mg/g), at the time (*t)*, *K1* and *K2* are the rate constant of the Pseudo -2nd -order kinetics sorption kinetics (min^–1^), the linear graph is drawn with *t/qt* and contact time (*t*) (min).

The biosorption of As^III^ on CASp2b supports the pseudo-2nd–order kinetics with the R^2^ value of 0.968 (Fig. 3b).The pseudo-2nd-order kinetics supports chemisorption as the mode of biosorption^89^. The pseudo 2nd-order kinetics have also been found suitable for the adsorption of copper, zinc, and cobalt on calcium alginate beads with *R*^2^ value of 0.999^76^. Therefore, it is suggested that this biosorption process might have occurred by chemical interaction between As^III^ in the solution and two surface active or binding sites on the biosorbent^29^.

#### Biosorption studies using CASp2b

##### Effect of initial As^III^ concentration and Isotherm studies

**Figure 4:**
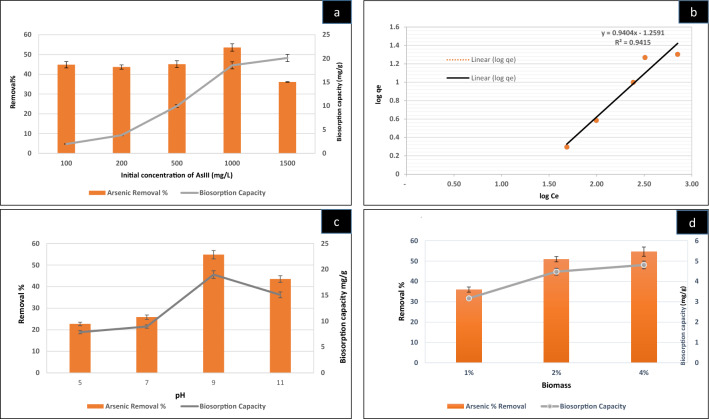
Biosorption studies of CASp2b (**a**) at variable initial concentration of As^III^ (**b**) Freundlich Isotherm (**c**) at variable pH (**d**) using variable biomass. The constant conditions of biosorption experimentations include (temp-35 °C; 120 rpm, time-20 min, pH-9).

The biosorption potential of CASp2b using variable initial As^III^ doses under optimal conditions is depicted in (Fig. 4a).The biosorption capacity of CASp2b increased with As^III^ dose from 100 to 1500 mg/L from 1.97 ± 0.07 to 20.1 ± 0.76 mg/g. The As^III^ removal percent from 100 to 1000 mg/L dose was 44.84 ± 1.56% to 53.53 ± 1.87%, followed by a decline to 36.12 ± 0.16 at the higher 1500 mg/L dose. A similar pattern of variability of biosorption concerning initial concentration has been reported in earlier studies^21,79^. Similarly, low biosorption % of As^III^ and As^V^ on bacterial biosorbent (*B*. *salmalaya* biomass) at a low dose (500 μg/L) and high at a higher dose (1000 μg/L) has been reported earlier^56^. This phenomenon occurs because of a reduction in the metal's resistance capacity in an aqueous solution with an increase in As^III^ concentration ^90,91^. At higher doses, the gradual decline in the availability of surface binding sites on the biosorbent might be responsible for reduction in biosorption^89–91^.

The equilibrium isotherms are used to describe the experimental adsorption data. These isotherms help predict the nature of biosorption by mutual sorbate and sorbent interaction. In this study, we have plotted the experimental data onto Langmuir and Freundlich isotherm models with their determination coefficient (R^2^). Regression value is essential in defining the suitability of kinetics and isotherms models for our experiments. The value of R^2^ is regarded as a measure of the goodness of fit of the experimental data to the isotherm models. The R^2^ value of a favorable linear isotherm graph can be in the range of 0–1, with a perfectly linear graph at 1; therefore, the best-fit isotherms are considered with values either one or close to one.

The Langmuir isotherm model mechanism assumed by the Langmuir isotherm is the sorption of the sorbate as a uniform or homogenous single layer. Once the sites are saturated, no further increase in sorption shall occur^92^. The Langmuir isotherm can be expressed in its linear form by the given Eq. (5)

5 $$\frac{{C}_{e}}{{q}_{e}}=\frac{1}{{q}_{max}K}+\frac{{C}_{e}}{{q}_{max}},$$

where (*Ce*) is the equilibrium concentration,(*K )*is the equilibrium constant, and *q*_*max*_ is the maximum absorption capacity. The Langmuir isotherm regression value (*R*^2^) obtained was 0.223 from the linear graph plotted between *(Ce/qe)* versus *Ce* (Supplementary Fig. 3).

Freundlich isotherm model: The Freundlich equation and graph (Fig. 4b) describe the heterogeneous nature of the adsorption process^93^. The Freundlich adsorption isotherm can be expressed in the linearized form^94^ by the following Eq. (6),

6 $${\text{log}}{q}_{e}={\text{log}}{k}_{f}+\frac{1}{n}{\text{log}}{C}_{e},$$

where *Ce* is the equilibrium concentration, (*k*_*f*_ ) and (*n*)are constant, which can evaluated from slopes and intercepts. The graph plotted between *log qe* versus *log Ce* in a linear equation.

The data of the concentration variability experiments under optimum conditions was computed in the respective isotherm equations to predict the most appropriate closely fitted equilibrium adsorption isotherm. After the computation of biosorption results, the R^2^ value was obtained, as shown in Table 1.The Freundlich isotherm was the best fit for CASp2b with R^2^ value of 0.941.The Freundlich isotherm is used for multilayer adsorption on heterogeneous sites with a two-parameter model^1,95^. Freundlich model is also suitable for other adsorption studies with different adsorbents and adsorbates^1^.

**Table 1:** Biosorption isotherm results of CASp2b.

Isotherms	Linear formula	Plots	R^2^	q_max_	Reference
Langmuir	$\frac{Ce}{qe}=\frac{1}{{q}_{m}}k+ \frac{Ce}{qm}$	(Ce/qe) versus Ce	0.2233	12.5	^92^
Freundlich	$log qe=log{k}_{f }+\frac{1}{n}log{c}_{e}$	log qe versus log Ce	0.9415	1.06	^94,115^

##### Effect of pH

Biosorption mainly depends on the sorbate’s chemical nature and the sorbent's surface functional groups, which rely on the pH of the solution^82,96^. Thus, pH is considered the most critical factor for estimating sorption capacity using any sorbent. The effect of pH variation on the biosorption efficiency of As^III^ on CASp2b was studied at variable pH 5, 7, 9, 11 (Fig. 4c), keeping the other factors constant (Biomass dose: 2 gm wet weight in 100 mL, contact time: 20 min, temperature: 35 ℃, NaAsO_2_ concentration: 1000 mg/L). The results of pH variation experiments showed increased biosorption capacity and As^III^ removal percent with increasing pH, with their peaks at pH 9 (18.99 ± 0.75 and 54.81 ± 1.91) followed by a decline at pH 11 (15.08 ± 0.57 and 43.53 ± 1.52).

The alkaline pH is also suitable (maximum at pH8) for the sorption of As^III^ on octahedral TiO_2_ nanocrystals^97^. The cation exchanger biosorbent developed by saponification of waste watermelon rind to the increase carboxyl functional groups also exhibited an increase in biosorption with an increase in pH upto pH 12^82^. The pH of the solution directly affects the chemical state of arsenic. As^III^ primarily exists as a neutral species (H_3_AsO_3_) in the acidic to a neutral range(pH 2–7) but exists as an anionic species (H_2_AsO_3_^–1^) and (HAsO_3_^–2^) in the alkaline pH range of 7–12^82^. These anionic species (H_2_AsO_3_^–1^ and HAs O_3_^–2^) of As at pH 9 might be responsible for better interaction with the biosorbent used in the present study.

##### Effect of biomass variation

The effect of biomass on biosorption capacity was studied using three different biomass percent(1%, 2%, and 4% wet biomass) with a constant As^III^ dose (200 mg/L),which is shown in (Fig. 4d).The highest arsenic removal percent and biosorption capacity was observed with 4% biomass (54.64 ± 2.26% and 4.81 ± 0.19) followed by 2% (50.92 ± 1.27% and 4.48 ± 0.17) and 1% (36.03 ± 1.27%, 3.17 ± 0.11), showing an increase in biosorption with the increase in biomass (CASp2b).This increase can be attributed to the rise in surface area available for sorption^56^. The minor difference in biosorption capacity (mg/g of biomass) with increasing biomass concentration might be due to the splitting of the concentration gradient^98^.

##### Effect of temperature variation and thermodynamics

**Figure 5:**
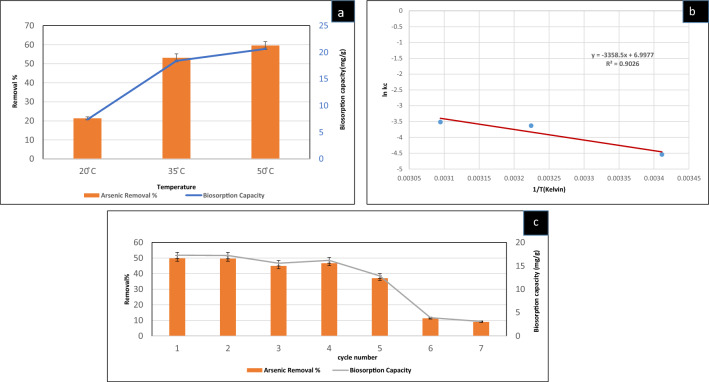
Biosorption studies for As^III^ using CASp2b (**a**) at variable Temperatures (20 °C, 35 °C, 50 °C); (**b**) Van’t Hoff plot of thermodynamics (**c**) Reusability of CA Sp2b beads in terms of As^III^ removal % and biosorption capacity in seven cycles (As^III^ − 1000 mg/L;pH 9; Biomass dose-2% wt/v; 20 min;35 °C; pH9; 120 rpm).

Temperature is considered an essential factor that affects the biosorption process tremendously. The effect of variable temperatures on the arsenic biosorption by CASp2b was examined under optimum experimental conditions with three temperature variations (20 ℃, 35 ℃, 50 ℃)and keeping the other factors constant(Biomass dose: 2 gm wet weight in 100 mL, contact time: 20 min, NaAsO_2_ concentration: 1000 mg/L). In our investigation, we noted a temperature-dependent rise in the removal percentage and biosorption capacity of As^III^, as outlined below: at 20 ℃ (R% = 21.33 ± 0.74, qe = 7.39 ± 0.03 mg/g), 35 ℃ (R% = 53.09 ± 2.12, qe = 18.4 ± 0.13 mg/g), and 50 ℃ (R% = 59.55 ± 2.08, qe = 20.64 ± 0.14 mg/g) for the biomass, respectively (Fig. 5a).

The observations are consistent with earlier studies^99,100^ with temperature dependent rise of biosorption due to rise in kinetic energy, decrease in liquid viscosity and increase in surface activity. Moreover, elevation of temperature promotes the rate of diffusion of metals or sorbent from outer to the inner layers through the porous surface due to decrease in liquid viscosity.

The results of temperature variation were used to estimate biosorption thermodynamics. The evaluation of the thermodynamics of the biosorption process is essential to find the applicability of arsenic removal in natural systems, as this is a temperature-dependent process^101^. Thermodynamics parameters like ΔG° (Gibb's free energy), ΔH° (enthalpy), and ΔS° (entropy) are indicators of the possible nature of metal adsorption by green adsorbents. The reaction is considered spontaneous with a negative value of ∆G° and non-spontaneous with a positive value of ∆G°^102^. In a study, adsorption of As^III^ ions by green adsorbent was spontaneous with negative ∆G˚ values at the studied temperatures^103^. The following equation was used to estimate the thermodynamics of the biosorption process^54,104,105^.

7 $$\Delta G=-RT{\text{ln}}{K}_{c},$$

8 $${K}_{c}=\frac{\Delta H^\circ }{RT}-\frac{\Delta S^\circ }{RT},$$

9 $$\Delta G^\circ =\Delta H^\circ -T^\circ\Delta S^\circ ,$$

where ΔG° is Gibb’s free energy, ΔH° is enthalpy, ΔS° is entropy, R = gas constant (8.314 J/mol K) and T is the temperature (K), Kc is the distribution constant. The values of ΔH° and ΔS° (Table 2) were determined from the slope and intercept of the graph plotted between *ln Kc* (Y-axis) versus *1/T* (X-axis) (Fig. 5b).

**Table 2:** Biosorption thermodynamics results of CASp2b.

T (k)	ΔG (kJ/mol)	ΔH (kJ/mol)	ΔS (J/mol)
293 k	− 16.99	27.42	58.1
308 k	− 17.99		
323 k	− 18.74		

In our study (Table 2), the values of ΔG°were negative at the studied temperatures (− 16.99, − 17.99, − 18.74 at 293 k, 308 k, 323 k respectively).The negative ΔG° values increased with temperature, suggesting the biosorption is spontaneous^106^. The positive ΔH° value shows the endothermic nature of the biosorption of As^III^ on CASp2b. The positive ΔS° value indicates increased randomness at the interface of the biosorbent and the As^III^ amended aqueous solution. This increased randomness can be due to ligand exchange reaction at the interface during biosorption^82^. The thermodynamics parameters show that the process favors the removal of arsenic ions by CASp2b. The arsenic removal process by using the modified biomass of watermelon rind was also found to be spontaneous with negative ΔG° values and positive ΔH° and ΔS° values^82^.

##### Experiment of reusability

The reusability of the biosorbent CASp2b was investigated and the outcomes for seven consecutive cycles are depicted in Fig. 5c. The analysis of biosorption capacity shows reasonable reusability of the CASp2b beads from first to the fifth reuse cycle (17.27 ± 0.65–12.84 ± 0.5 mg/g). However, a notable decline occurred in the sixth and seventh cycles to 3.91 ± 0.13 and 3.11 ± 0.11 mg/g. Other workers have reported a similar “biosorption” reusability pattern^55,107^. This indicated suitability of the regeneration process upto five cycles.

##### SEM–EDX mapping of CASp2b

**Figure 6:**
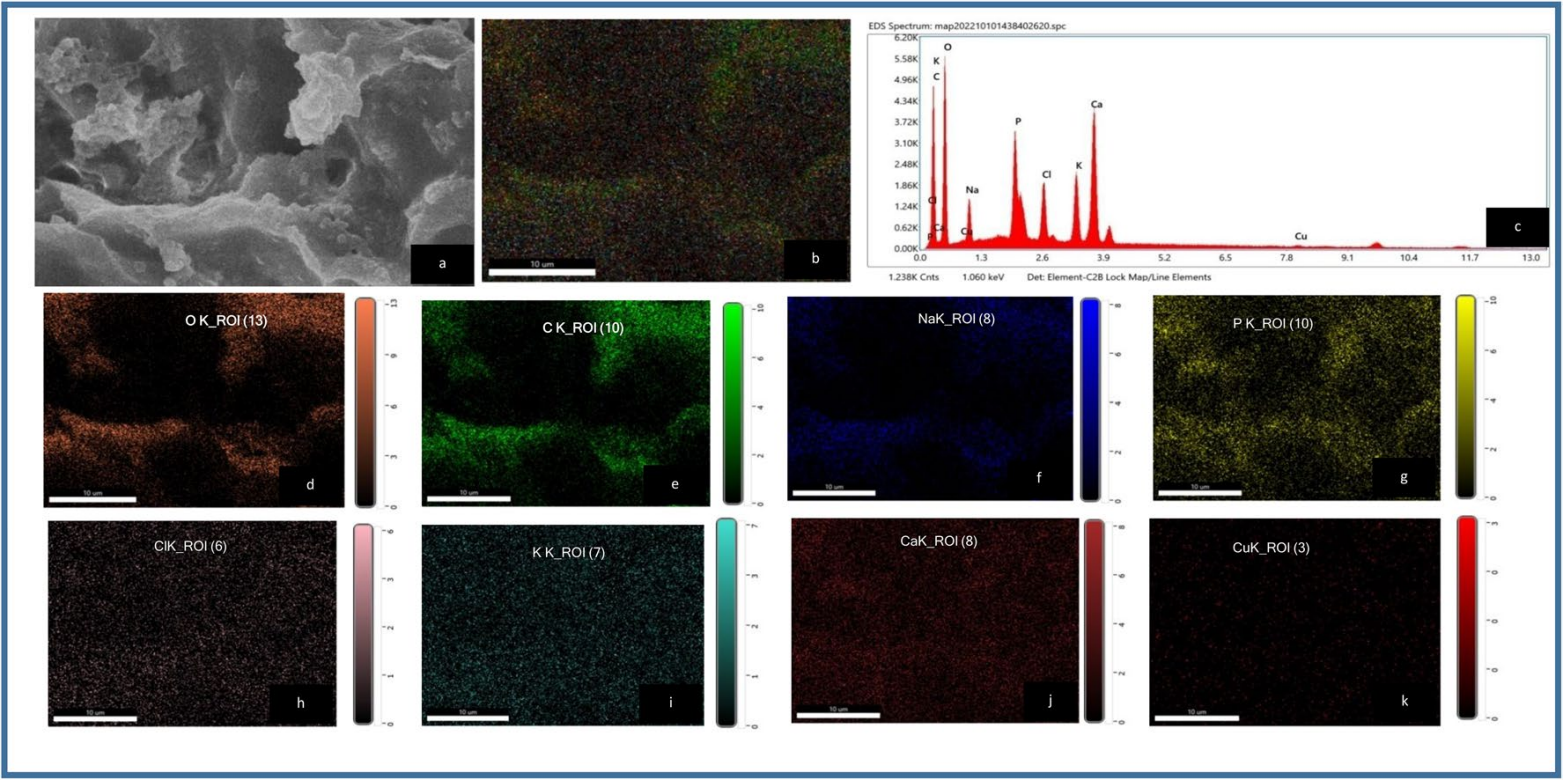
Energy dispersive spectroscopy (EDX) analysis of As^III^ unexposed CASp2b beads (**a**) SEM area under EDX analysis; (**b**) EDX mapping images of all overlapping elements. (**c**) EDX spectrum analysis of showing elemental composition and Elemental maps of (**d**) Oxygen (**e**) Carbon (**f**) Sodium (**g**) Phosphorous (**h**) Chlorine (**i**) Potassium (**j**) Calcium (**k**) Copper (Original figures are presented as Supplementary Fig. 4).

**Figure 7:**
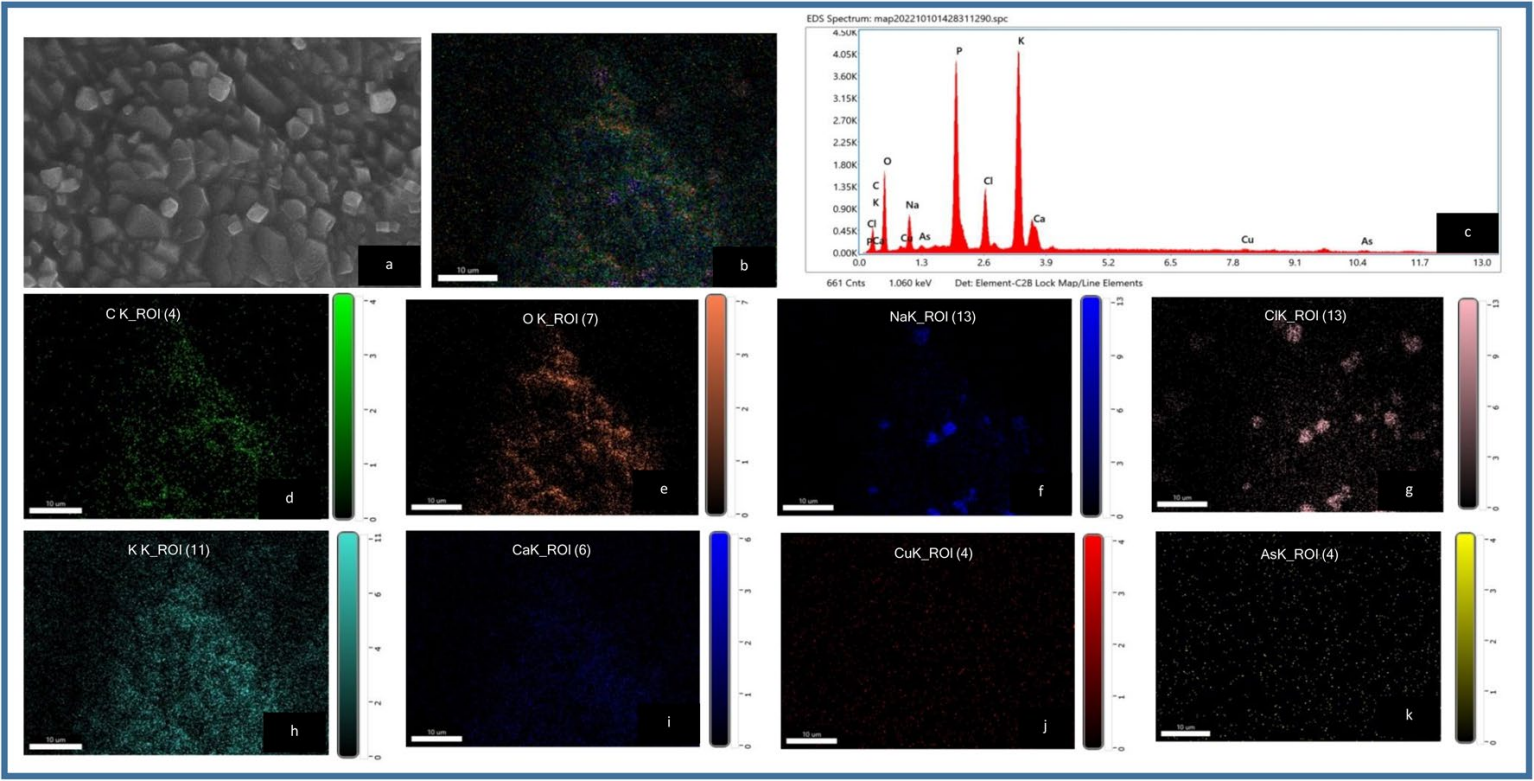
Energy dispersive spectroscopy (EDX) analysis of As^III^ exposed CASp2b biomass beads (**a**) SEM area under EDX analysis; (**b**) EDX mapping images of all overlapping elements. (**c**) EDX spectrum analysis showing elemental composition and Elemental maps of (**d**) Carbon (**e**) Oxygen (**f**) Sodium (**g**) Chlorine (**h**) Potassium (**i**) Calcium (**j**) Copper (**k**) Arsenic (Original figures are presented as Supplementary Fig. 5).

SEM was applied to examine the surface morphological characteristics of the biosorbent(CASp2b)and chemical characteristics were analyzed with EDX graphs and mapping before (Fig. 6) and after Arsenic biosorption (Fig. 7).The elemental mapping and EDX spectra provide clear evidence of As^III^ biosorption on CASp2b beads, as shown in Table 3.The EDX and mapping analysis shows the presence of elements like Carbon (C), Oxygen (O), Sodium (Na), Phosphorous(P), Chlorine (Cl), Potassium (K), Calcium (Ca), Copper (Cu) and Arsenic (As).The elemental composition (wt % and atomic %) was variable in the Arsenic unexposed and exposed group. The presence of sodium and arsenic on the surface of the arsenic-exposed group indicates surface biosorption of sodium and arsenic derived from sodium-meta-arsenite, which was added in the batch experimentations ).

**Table 3:** Elemental composition of arsenic unexposed and exposed CASp2b beads.

Elements	Arsenic unexposed CASp2b beads		Arsenic exposed CASp2b beads	
	Weight %	Atomic %	Weight %	Atomic %
C	29.6	41.9	6.7	13.1
O	40.3	42.9	30.9	45.2
Na	4.8	3.6	5.8	5.9
P	5.9	3.2	17.1	12.9
Cl	3.3	1.6	6.1	4.0
K	4.6	2.0	25.5	15.3
Ca	10.9	4.6	3.6	2.1
Cu	0.6	0.2	1.3	0.5
As	ND*	ND*	2.9	0.9

*ND  not detected.

These results imply that CASp2b consisting of As^III^-tolerant *Acinetobacter* bacterial species showcased remarkable biosorption with applicability at even elevated As^III^ concentrations, achieving over 35% As^III^ removal even at a high concentration of 1500 mg/L of As^III^. Notably, at an elevated temperature of 50 °C, the biosorption exceeded 55% As^III^ removal. Additionally, it was observed that CASp2b showcased significant potential for reusability.

### Comparison of CASp2b with other As^III^ biosorbent

The comprehensive findings emphasize the diverse potential of various biomaterials and microbes for biosorption, especially in removing As^III^ from waste water. Table 4 presents the maximum adsorption capacity (q_max_) and optimal conditions for As^III^ across various adsorbents explored in the literature alongside CASp2b^81,108–111^. Different biomasses of bacteria, fungi, algae, or plants have been tested in untreated dried or carbonized states for biosorption. Other workers have immobilized the biomass with metals like Fe, Mn, La to increase the As^III^ binding efficiency.

**Table 4:** Comparison of CASp2b with other As^III^ biosorbents.

Biosorbent	As^III^ Biosorption Potential					Ref
Live/dead biomass/immobilized	Optimum conditions			Maximum biosorption capacity(q_max_) (mg/g)	Reusability	
	pH	Temp	Time			
CASp2b	9	35 °C	20 min	20.1 mg/g w.wt	5 cycles	Present study
l-Histidine immobilized montmorillonite	6	44 °C	30 min	87.7 mg/g	Not mentioned	^116^
Immobilized biomass of *Garcinia cambogia*	6–8	–	30 min	–	5 cycles	^117^
La(III) loaded carboxyl functionalized watermelon rind	7.1	34.85 °C	–	62.50 + 0.11 mg/g	Several cycles	^82^
Iron impregnated fungal bio-filter (IIFB) discs of luffa sponge containing *Phanerochaete chrysosporium* mycelia	7	28 °C	30 min	92.4 mg/g	4 cycles	^81^
MPAC-500 and MPAC-600 (magnetic-activated carbons synthesized from the peel of *Pisum sativum* pea)	–	25 °C		0.7297 mg/g and 1.3335 mg/g, respectively	5 cycles	^111^
Dead algae *Chlamydomonas* sp.	4	25 °C	60 min	58.8 mg/g	5 cycles	^38^

Upon comparison, the biosorption capacity of CASp2b with other biosorbents exhibits moderate efficiency, considering wet-weight biomass for estimation in the current study. The calcium alginate immobilization of biomass has been applied for the removal of various metals like Ni, Pb, Zn^112^ and Cr, Pd^II^ ^113^ with high efficiency of metal removal, but evidence of As^III^ removal is rare. Consequently, the As^III^ biosorbent CASp2b distinguishes itself through its unique methods of preparation, utilization in wet biomass state, optimal biosorption conditions, and comparable efficiency in As^III^ removal, as presented in Table 4.

Biosorption is a complex process involving the chemical nature of the sorbate sorbent and other factors. The sorption behavior of heavy metals on biosorbent mainly depends on the oxidation state of metal and the surface functional groups on the biosorbent and the interplay of physical and chemical interactions between them^61,114^. Biosorption is impacted by many factors, such as pH, the simultaneous presence of other metals, the kind of biosorbent material, and many more^40,82,96,97^. The study’s results indicate the As^III^ biosorption potential of CASp2b, which can be used as an alternative to reduce arsenic contamination in water.

In the present study, the prime variables that affect the biosorption process, such as temperature, pH, biomass, and initial sorbate concentration, have been studied for the developed sorbent CASp2b. However, the efficacy of this biosorbent in natural conditions with the interference of other salts, metals, etc., needs to be explored in further studies. In earlier investigations^40,42^, the biosorption capabilities of *Acinetobacter* species in the presence of multiple metals, including Cd, Cr^VI^, Cu^II^, and Ni^II^. The research findings indicate that beyond the metals mentioned, *Acinetobacter* exhibits potential as a viable biosorbent for As^III^. Our study aligns with other investigations focused on As^III^ biosorption using microbes. Although our *Acinetobacter* strain demonstrates a lower q_max_ of 20.1 mg/g compared to other studies, noteworthy characteristics include its effectiveness at high concentrations (1500 mg/l) and elevated temperatures, with substantial reusability. Therefore, *Acinetobacter* emerges not only as a proficient biosorbent for As^III^ removal from contaminated water but also holds promise as a prospective option for removing other heavy metals in the future.

## Conclusion

This study compiles the isolation and development of an As^III^ biosorbent using the biomass of bacterial isolate Sp2b.This bacterium Sp2b showed hyper-tolerance to As^III^ with MIC of 7 g/L (53.88 mM). It was designated as *Acinetobacter* sp. strain Sp2b per the sequencing, biochemical, and staining results.On comparing the biosorption capacity of the calcium alginate immobilized dead bacterial biomass (CASp2b) to the live immobilized biomass, it was discovered that both demonstrated better biosorption efficiency than the unimmobilized (biomass alone) biomass. The application of the CASp2b in a batch reaction system with the advantage of easy removal, reusability, and enlargement of surface area with higher metal affinity was conducted using variables of pH, initial As^III^ concentration, biomass, and temperature to obtain optimum biosorptive conditions. This batch of experimental systems using the CASp2b exhibited encouraging biosorption potential. At the optimum conditions of biosorption with 35 °C, pH 9, using 2gm wet weight in 100 mL with an agitation speed of 120 rpm within 20 min of contact time, using 1000 mg/L of initial As^III^ concentration, the removal percentage of As^III^ was 53.53%, and a maximum biosorption capacity (q_max_) of 20.1 ± 0.76 mg/g was obtained. The isotherm and kinetic modeling of the experimental data favored Freundlich isotherm (R^2^ = 0.941) and pseudo-2nd-order kinetics (R^2^ = 0.968).The biosorption analysis data of CASp2b fitted best in the Freundlich isotherm instead of the Langmuir isotherm, indicating the multiplayer sorption and chemisorption as dominant adsorption mechanisms as per the pseudo-2nd-order kinetics.

The thermodynamic study suggested the spontaneous and endothermic nature of this biosorption process with negative ΔG° values at all the temperatures along with positive enthalpy (ΔH ° = 27.42) and entropy (ΔS° = 58.1). These results indicate the potential application of CASp2b for the remediation of arsenic-contaminated water with reasonable reusability to reduce the arsenic-generated toxic effects. The research represents a valuable contribution to understanding arsenic biosorption materials by employing immobilized Sp2b biomass beads. These beads have demonstrated the ability to efficiently eliminate elevated concentrations of As^III^ from aqueous solutions, particularly at moderate to high temperatures.

### Supplementary Information

Supplementary Information.

## Data Availability

The data represented in the manuscript is the experimental data generated by the authors, which shall be made available on demand from the corresponding author. The 16S rDNA sequence of Sp2b has been deposited at NCBI with OP010048 accession number.
