# Supplementary material for: Biosorption of arsenic (III) from aqueous solution using calcium alginate immobilized dead biomass of Acinetobacter sp. strain Sp2b
Source: Sci Rep. 2024 Apr 30;14:9972. doi: 10.1038/s41598-024-60329-7 (PMC11063054; doi:10.1038/s41598-024-60329-7)
Supplement: Supplementary file 1 — Supplementary Information. [file 41598_2024_60329_MOESM1_ESM.docx]

**Supplementary Documents**

**Supplementary Figure 1**:Agarose gel electrophoresis First Lane(L) is of DNA 500bp ladder (500bp-5000 bp) and the second lane consists of 16 S rDNA PCR product.


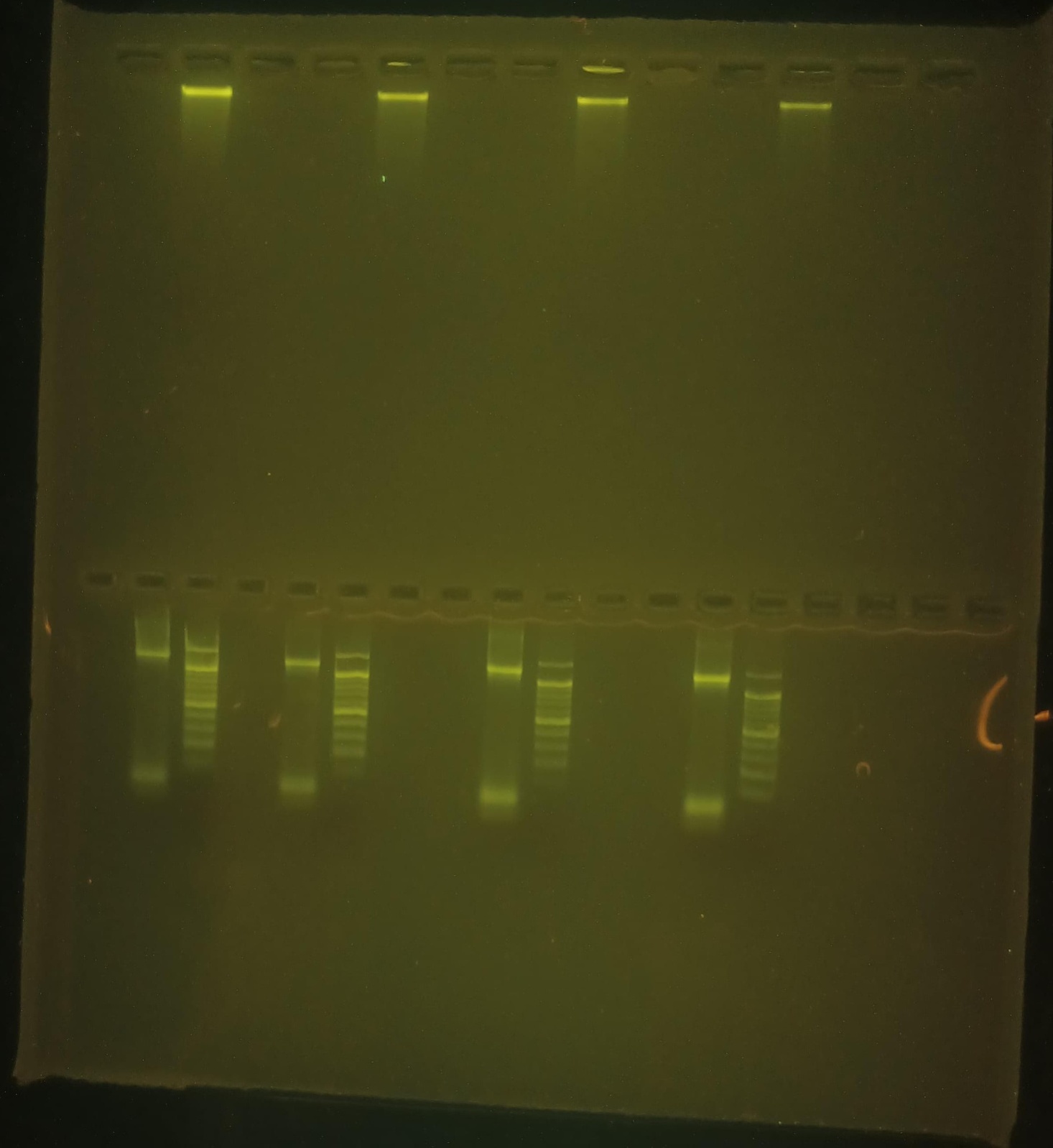


Genomic DNA of other bacteria

Genomic DNA of Sp2b

Amplified fragment of other isolated bacteria

Amplified fragment of Sp2B

**Supplementary Figure 2:-**Pseudo -1^st^-order kinetic plot of As^III^ biosorption from aqueous solution using CASp2b (Time-5-120 min, pH- 9;Biomass dose- 2% wt/v ;AsIII- 200mg/L ;35˚C;120rpm)

**Supplementary Figure 3:-**Langmuir Isotherm plot for biosorption of As^III^ onto CASp2b (pH 9; Biomass dose- 2%wt/v; residence time: 20 min; temperature 35 ̊C)

**Supplementary Figure 4 :-**Raw data of Scanning Electron Microscope- Energy dispersive spectroscopy (EDX) analysis of As ^III^ unexposed CA Sp2b beads. It was performed by FESEM-JEOL India Pvt. Ltd, Model number-JSM7610FPLUS, at Manipal University, Jaipur. (A) It is showing SEM area under EDX analysis; (B) EDX mapping images of all overlapping elements (C) Elemental maps of Carbon, Oxygen, Sodium, Phosphorous, Chlorine , Potassium , Calcium , Copper; (D) EDS spectrum

**S.Fig.4 (A)**

|  | | | | | | |
| --- | --- | --- | --- | --- | --- | --- |
|  | | | | | | |
|  | 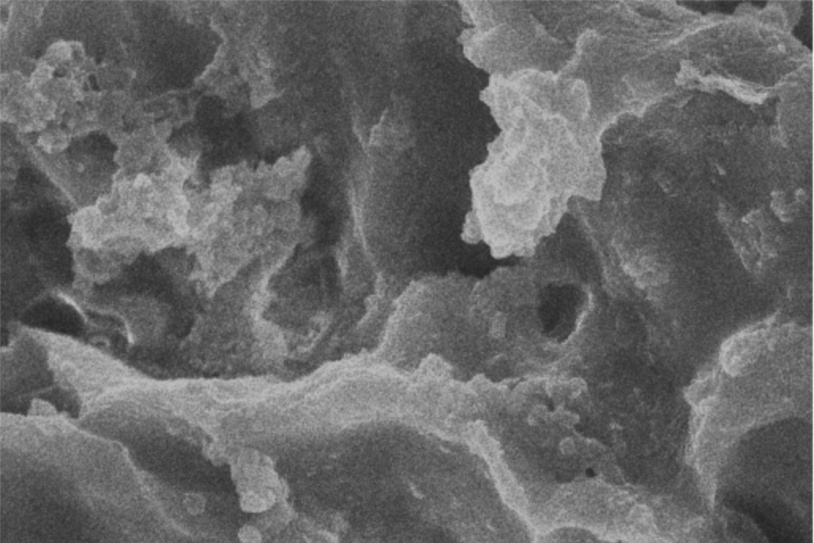 |  |  |  | | |
|  |  |  | | | | |
|  |  |  | | | | |
|  |  |  | | | | |
| **S.Fig.4 (B)** | | | | | |  |
|  | | | | | | |
|  | 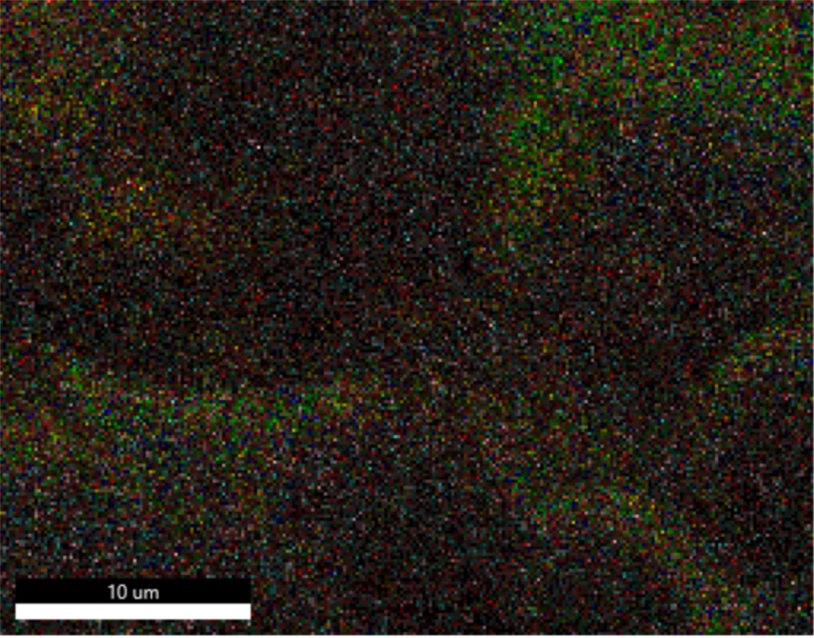 |  | 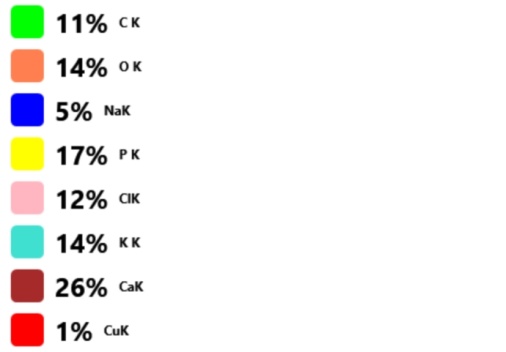 |  |  |  |
|  |  |  |  |  |  |  |
|  |  |  |  |  | | |
|  |  |  | | | | |
|  |  |  | | | | |
|  | | | | | | |

**S.Fig.4 (C)**

|  | | | | | |  | |  |
| --- | --- | --- | --- | --- | --- | --- | --- | --- |
|  | | | | | | | | |
|  | 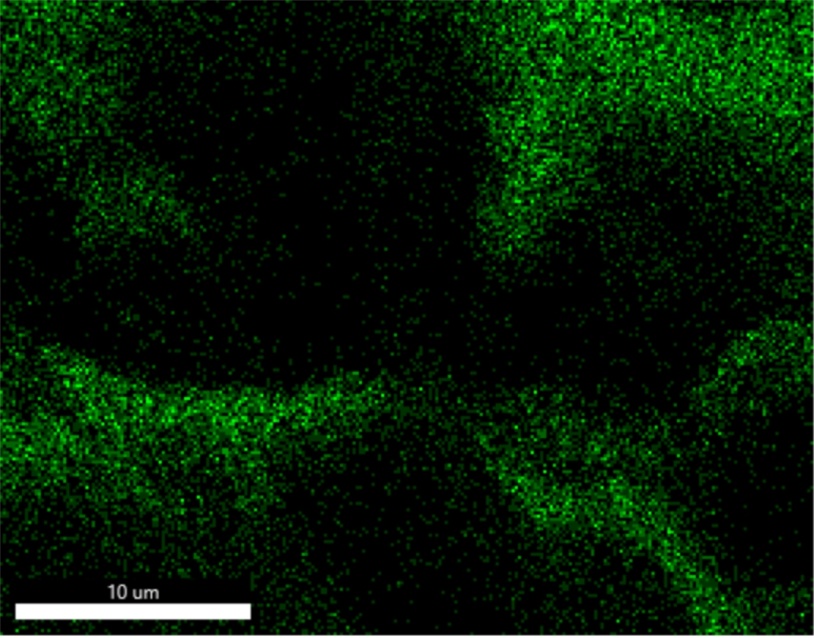 |  | C K_ROI (10) | | | |  | |
|  |  |  | | | | | | |
|  |  |  | | 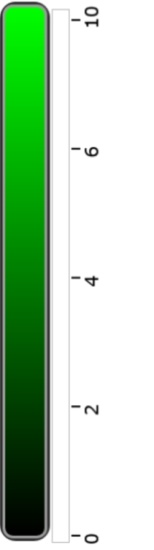 |  | | | |
|  |  |  | |  |  | | | |
|  |  |  | |  |  | | | |
|  | | | | | | | | |
|  | 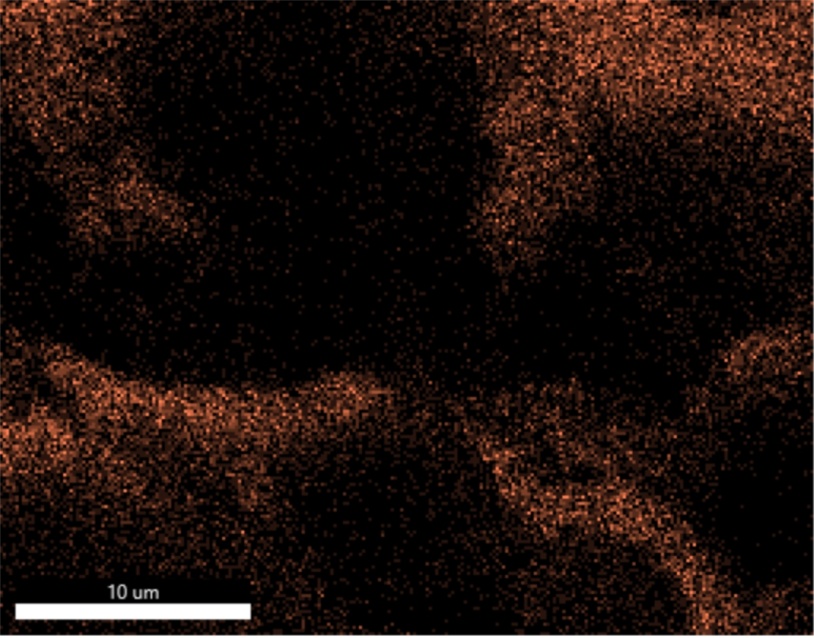 |  | O K_ROI (13) | | | |  | |
|  |  |  | | | | | | |
|  |  |  | | 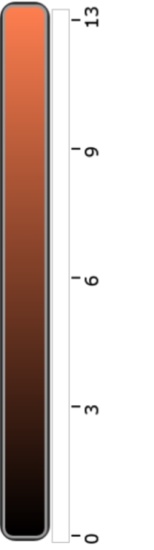 |  | | | |
|  |  |  | |  |  | | | |
|  |  |  | |  |  | | | |
|  | | | | | | | | |
|  | 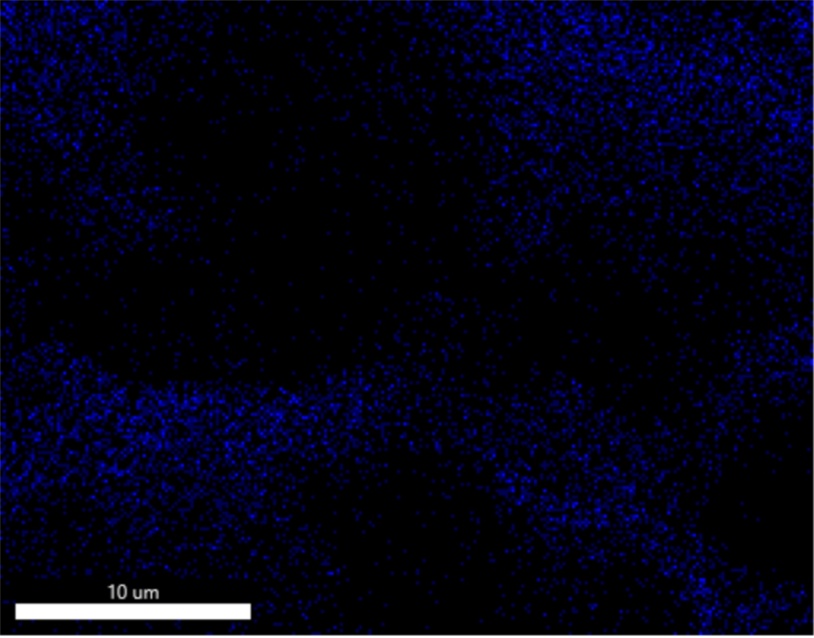 |  | NaK_ROI (8) | | | |  | |
|  |  |  | | | | | | |
|  |  |  | | 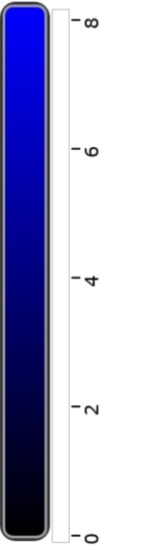 |  | | | |
|  |  |  | |  |  | | | |
|  |  |  | |  |  | | | |
|  | | | | | | | | |
|  | | | | | | | | |

|  | | | | | | |  | | |  | |
| --- | --- | --- | --- | --- | --- | --- | --- | --- | --- | --- | --- |
|  | | | | | | |  |  |  |  | |
|  | | | | | | | | | | | |
|  | 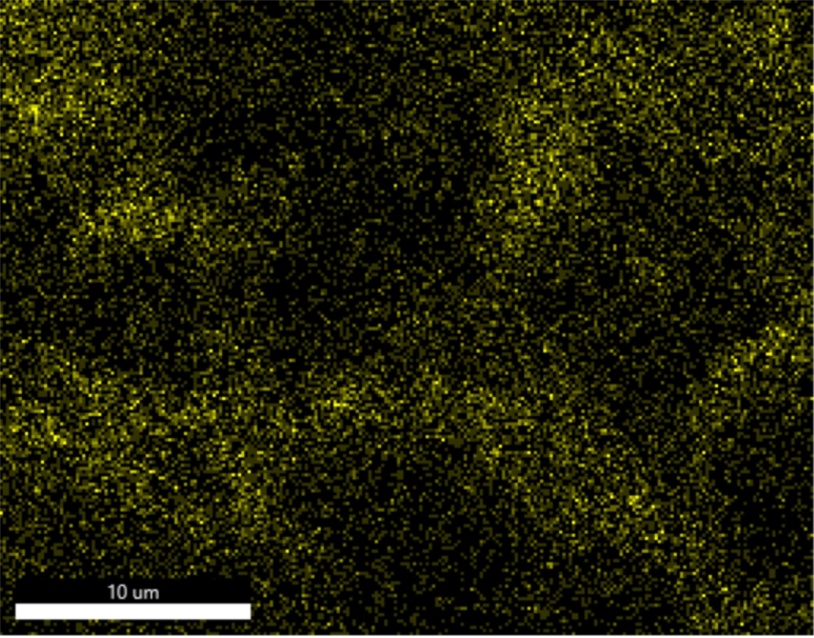 | |  | P K_ROI (10) | | | |  | | | |
|  |  |  |  | | | | | | | | |
|  |  |  |  | | 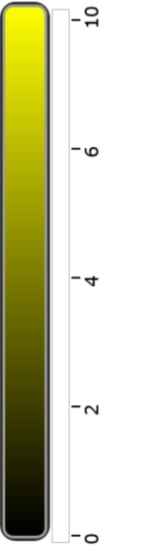 |  | | | | | |
|  |  |  |  | |  |  | | | | | |
|  |  |  |  | |  |  | | | | | |
|  | | | | | | | | | | | |
|  | 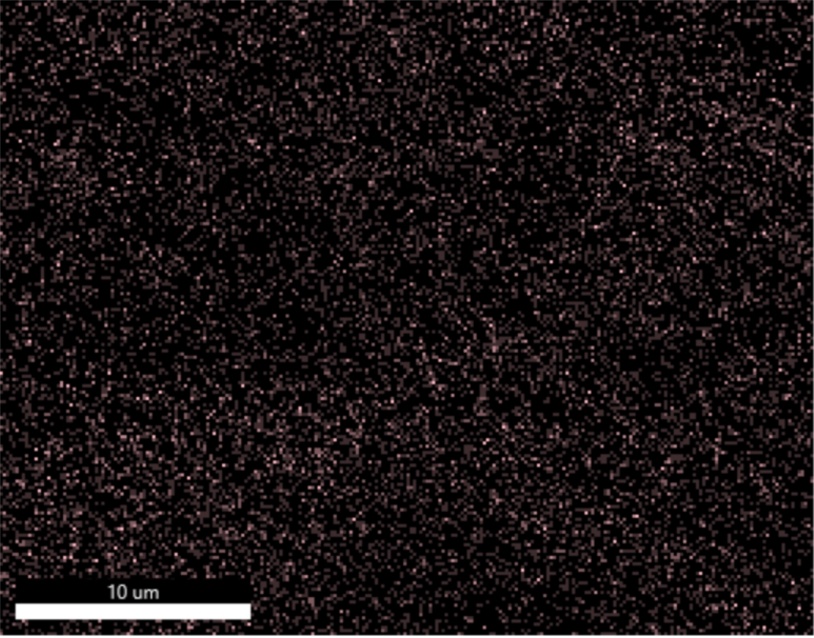 | |  | ClK_ROI (6) | | | |  | | | |
|  |  |  |  | | | | | | | | |
|  |  |  |  | | 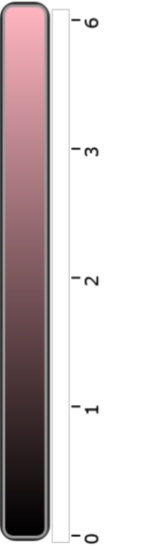 |  | | | | | |
|  |  |  |  | |  |  | | | | | |
|  |  |  |  | |  |  | | | | | |
|  | | | | | | | | | | | |
|  | 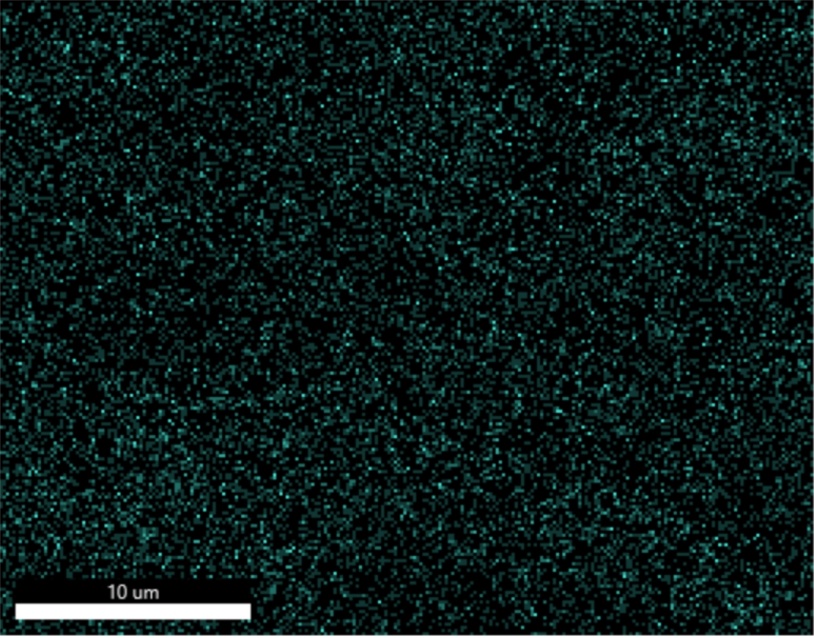 | |  | K K_ROI (7) | | | |  | | | |
|  |  |  |  | | | | | | | | |
|  |  |  |  | | 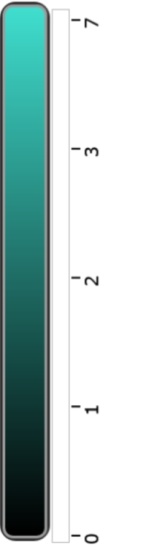 |  | | | | | |
|  |  |  |  | |  |  | | | | | |
|  |  |  |  | |  |  | | | | | |
|  | | | | | | | | | | | |
|  | | | | | | | | | | | |
|  | |  | | | | | | |  | |  |

|  | | | | | | |  | | |  | |
| --- | --- | --- | --- | --- | --- | --- | --- | --- | --- | --- | --- |
|  | | | | | | |  |  |  |  | |
|  | | | | | | | | | | | |
|  | 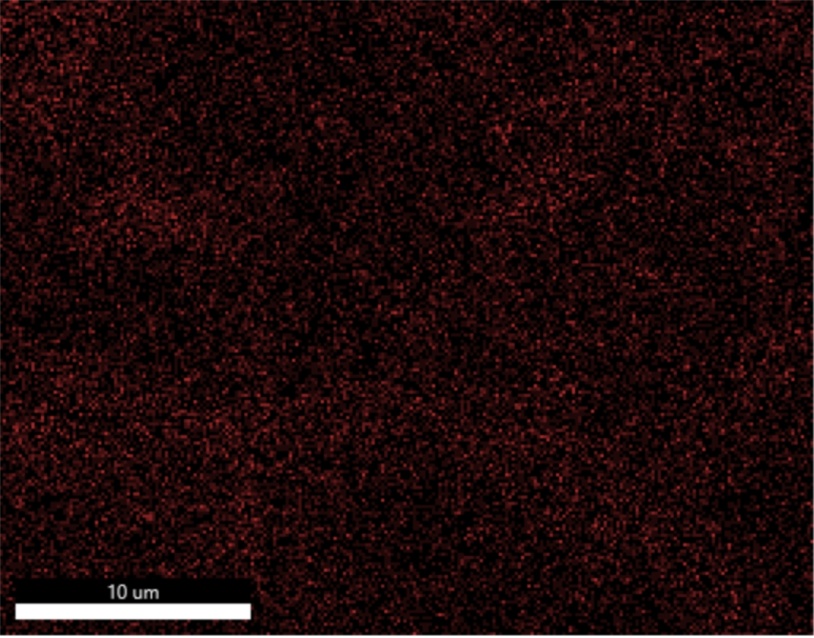 | |  | CaK_ROI (8) | | | |  | | | |
|  |  |  |  | | | | | | | | |
|  |  |  |  | | 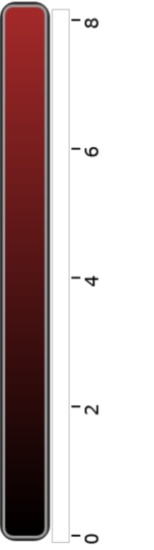 |  | | | | | |
|  |  |  |  | |  |  | | | | | |
|  |  |  |  | |  |  | | | | | |
|  | | | | | | | | | | | |
|  | 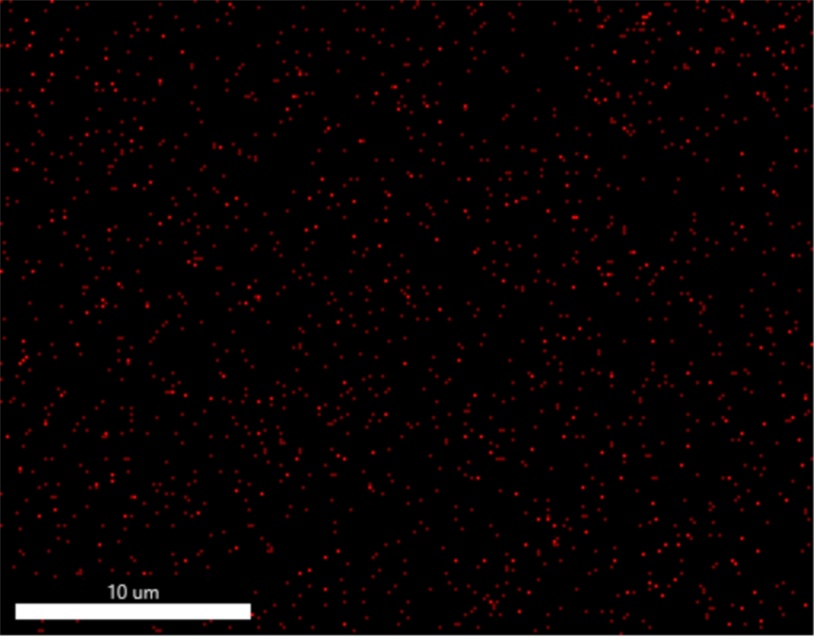 | |  | CuK_ROI (3) | | | |  | | | |
|  |  |  |  | | | | | | | | |
|  |  |  |  | | 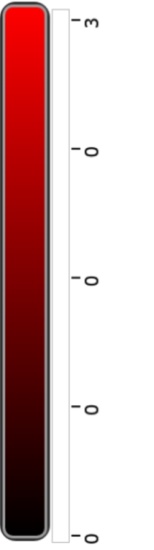 |  | | | | | |
|  |  |  |  | |  |  | | | | | |
|  |  |  |  | |  |  | | | | | |
|  | | | | | | | | | | | |
|  | | | | | | | | | | | |
|  | | | | | | | | | | | |
|  | | | | | | | | | | | |
|  | | | | | | | | | | | |
|  | |  | | | | | | |  | |  |

**S.Fig.4 (D)**

|  | | | | | | | | | | | | | |  | | | |  | | |
| --- | --- | --- | --- | --- | --- | --- | --- | --- | --- | --- | --- | --- | --- | --- | --- | --- | --- | --- | --- | --- |
|  | | | | | | | | | | | | | |  |  |  |  |  | | |
|  | | | | | | | | | | | | | | | | | | | |  |
|  | | | | | | | | | | | | | | | | | | | |  |
| kV: | 20 | Mag: | 3700 | Takeoff: | | 32.6 | |  | Live Time(s): | | 163.7 | Amp Time(µs): | | | | 3.84 | Resolution:(eV) | | 130 |  |
|  | | | | | | | **Sum Spectrum** | | | | | | |  | | | | | |  |
| 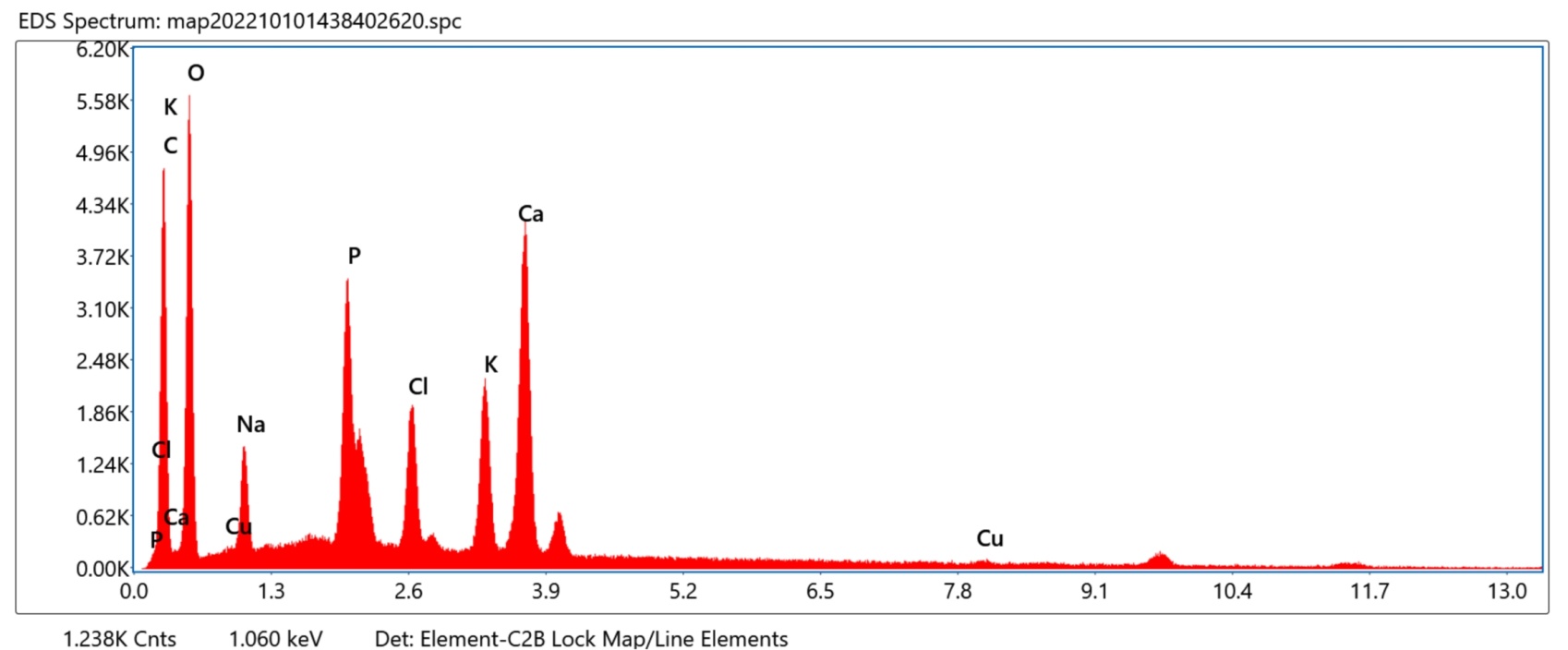 | | | | | | | | | | | | | | | | | | | |  |
|  |  |  |  |  |  |  |  |  |  |  |  |  |  |  |  |  |  |  |  |  |
|  |  |  |  |  |  |  |  |  |  |  |  |  |  |  |  |  |  |  |  |  |
|  |  |  |  |  |  |  |  |  |  |  |  |  |  |  |  |  |  |  |  |  |
|  | | | | | | | | | | | | | | | | | | | | |
| **Smart Quant Results** | | | | | | | | | | | | | | | | | | | |  |
|  | | | | | Element | | | | Weight % | Atomic % | | Error % |  | | | | | | | |
|  | | | | | C K | | | | 29.6 | 41.9 | | 9.1 |  | |  | | | | | |
|  | | | | | O K | | | | 40.3 | 42.9 | | 10.1 |  | |  | | | | | |
|  | | | | | NaK | | | | 4.8 | 3.6 | | 9.6 |  | |  | | | | | |
|  | | | | | P K | | | | 5.9 | 3.2 | | 3.3 |  | |  | | | | | |
|  | | | | | ClK | | | | 3.3 | 1.6 | | 3.2 |  | |  | | | | | |
|  | | | | |  |  |  |  |  |  |  |  |  |  |  | | | | | |
|  | | | | | K K | | | | 4.6 | 2.0 | | 3.0 |  | |  | | | | | |
|  | | | | | CaK | | | | 10.9 | 4.6 | | 2.3 |  | |  | | | | | |
|  | | | | | CuK | | | | 0.6 | 0.2 | | 14.7 |  | |  | | | | | |
|  | | | | | | | | | | | | | | | | | | | | |
|  | | | | | | | | | | | | | | | | | | | | |

**Supplementary Figure 5:-**Raw data of Scanning Electron Microscope- Energy dispersive spectroscopy (EDX) analysis of As ^III^ exposed CASp2b biomass beads. It was performed by FESEM-JEOL India Pvt. Ltd, Model number-JSM7610FPLUS, at Manipal University, Jaipur. It is showing (A) SEM area under EDX analysis; (B) EDX mapping images of all overlapping elements; (C) Elemental maps of Carbon , Oxygen, Sodium, Chlorine, Potassium, Calcium, Copper, Arsenic; (D) EDS spectrum

**S.Fig.5 (A)**

|  | | | | | | |
| --- | --- | --- | --- | --- | --- | --- |
|  | | | | | | |
|  | 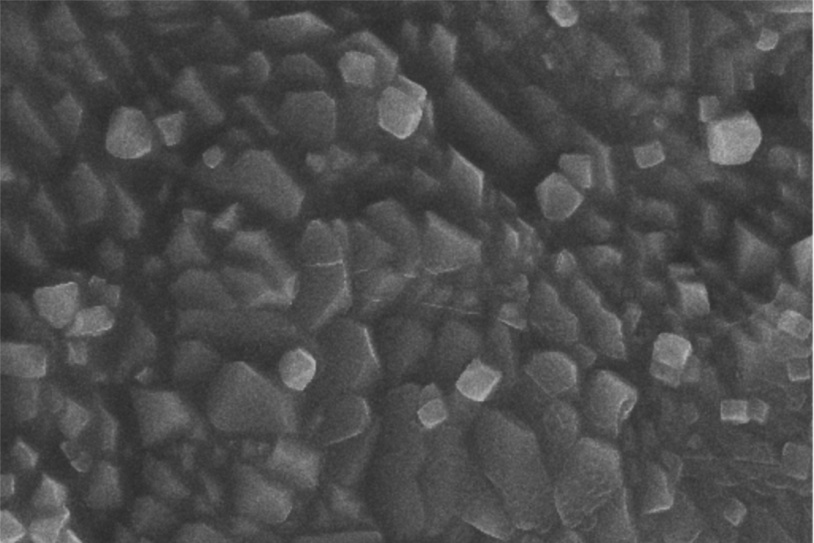 |  |  |  | | |
|  |  |  | | | | |
|  |  |  | | | | |
|  |  |  | | | | |
| **S.Fig.5 (B)** | | | | | |  |
|  | | | | | | |
|  | 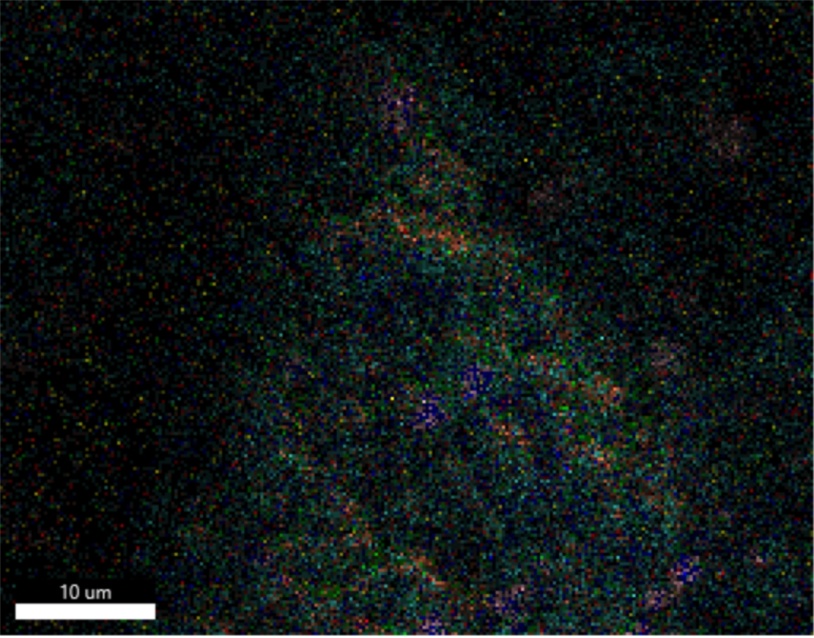 |  | 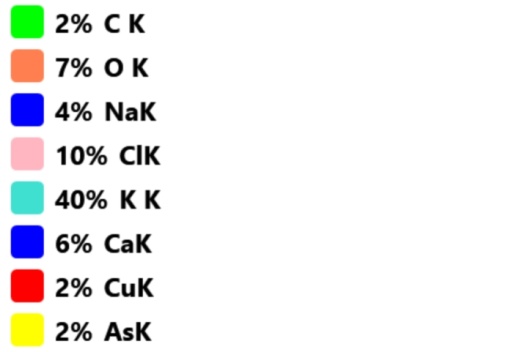 |  |  |  |
|  |  |  |  |  |  |  |
|  |  |  |  |  | | |
|  |  |  | | | | |
|  |  |  | | | | |
|  | | | | | | |

| \|  \| **S.Fig.5 (C)** \| \| --- \| --- \| | | | | | |  | |  |
| --- | --- | --- | --- | --- | --- | --- | --- | --- | --- | --- |
|  | | | | | |  |  |  |
|  | | | | | | | | |
|  | 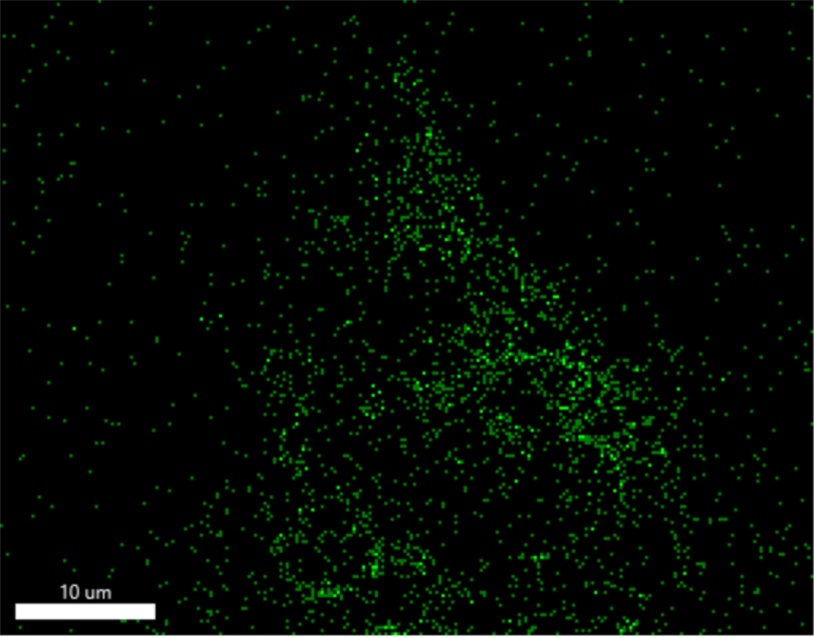 |  | C K_ROI (4) | | | |  | |
|  |  |  | | | | | | |
|  |  |  | | 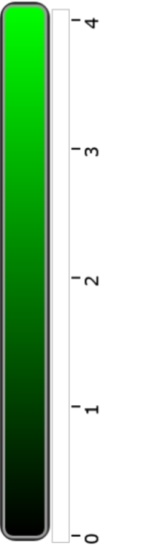 |  | | | |
|  |  |  | |  |  | | | |
|  |  |  | |  |  | | | |
|  | | | | | | | | |
|  | 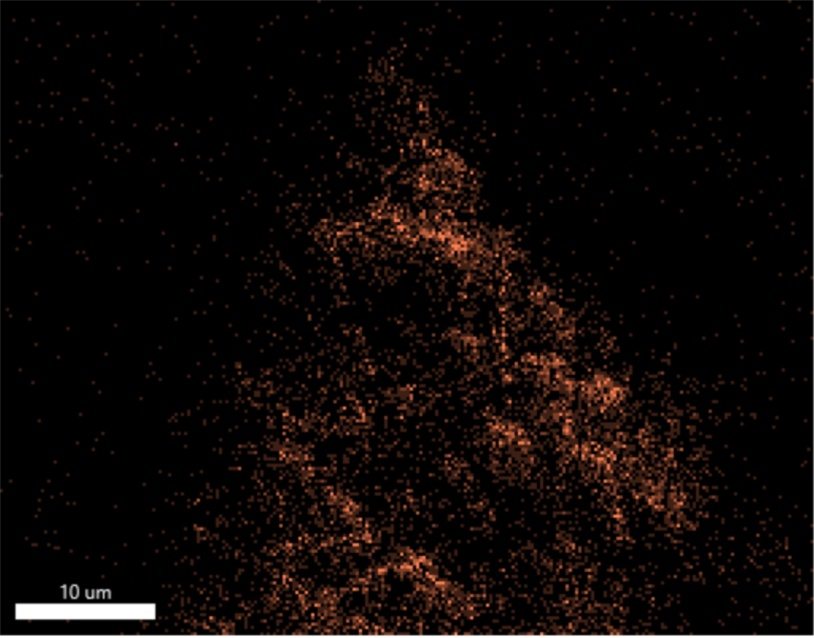 |  | O K_ROI (7) | | | |  | |
|  |  |  | | | | | | |
|  |  |  | | 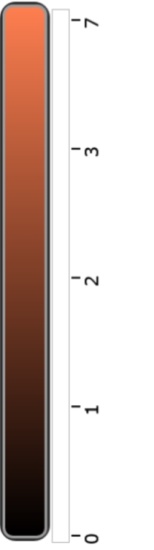 |  | | | |
|  |  |  | |  |  | | | |
|  |  |  | |  |  | | | |
|  | | | | | | | | |
|  | 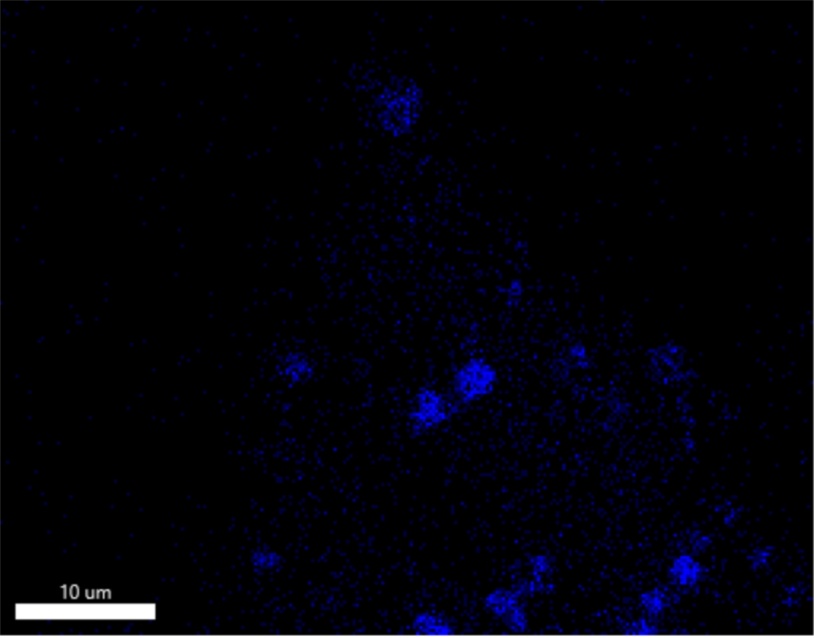 |  | NaK_ROI (13) | | | |  | |
|  |  |  | | | | | | |
|  |  |  | | 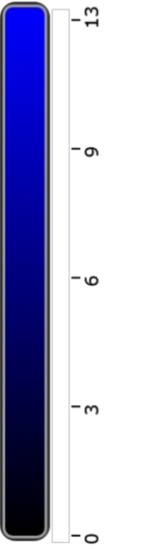 |  | | | |
|  |  |  | |  |  | | | |
|  |  |  | |  |  | | | |
|  | | | | | | | | |
|  | | | | | | | | |

|  | | | | | |  | |  |
| --- | --- | --- | --- | --- | --- | --- | --- | --- |
|  | | | | | |  |  |  |
|  | | | | | | | | |
|  | 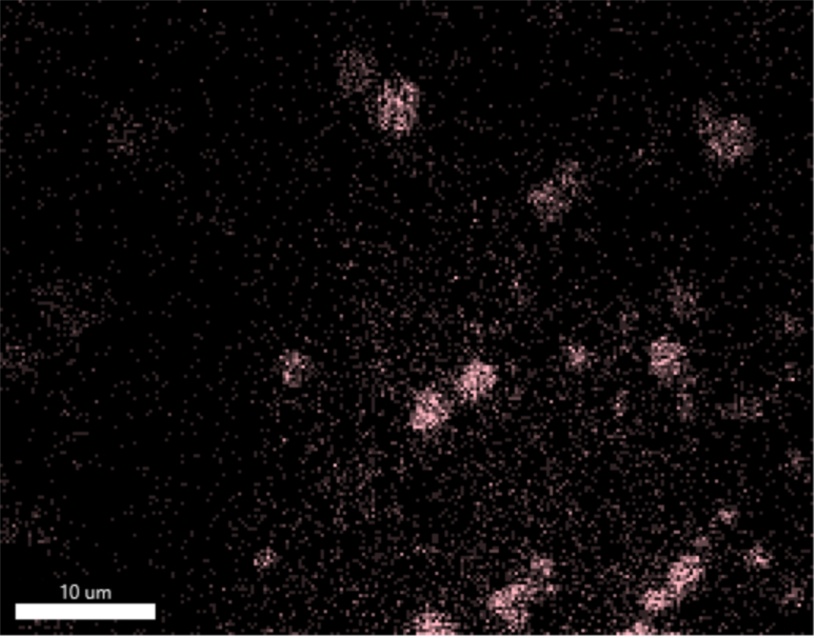 |  | ClK_ROI (13) | | | |  | |
|  |  |  | | | | | | |
|  |  |  | | 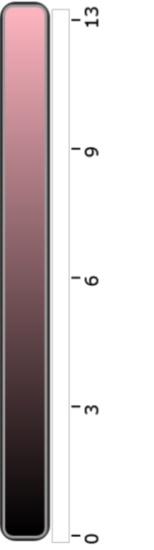 |  | | | |
|  |  |  | |  |  | | | |
|  |  |  | |  |  | | | |
|  | | | | | | | | |
|  | 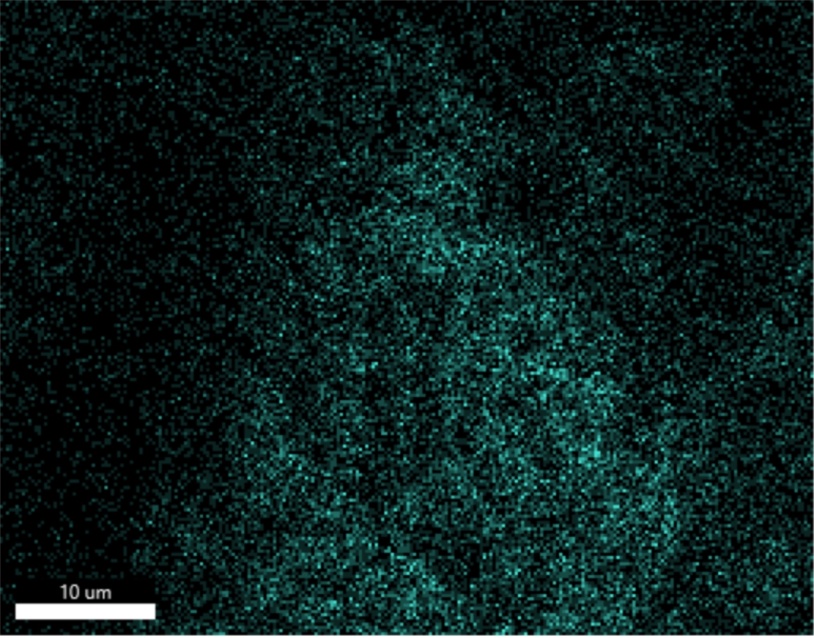 |  | K K_ROI (11) | | | |  | |
|  |  |  | | | | | | |
|  |  |  | | 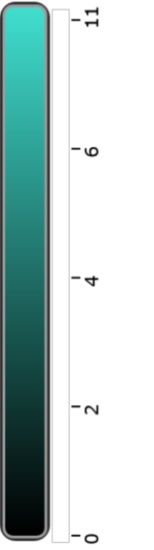 |  | | | |
|  |  |  | |  |  | | | |
|  |  |  | |  |  | | | |
|  | | | | | | | | |
|  | 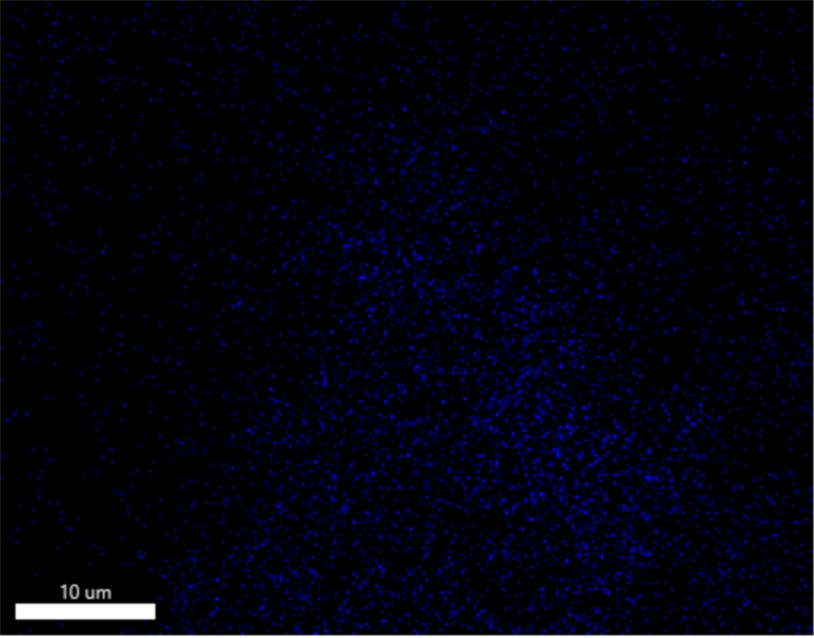 |  | CaK_ROI (6) | | | |  | |
|  |  |  | | | | | | |
|  |  |  | | 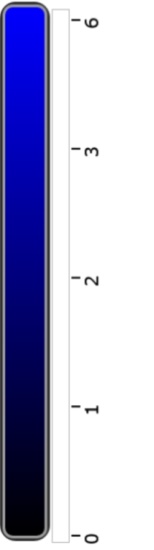 |  | | | |
|  |  |  | |  |  | | | |
|  |  |  | |  |  | | | |
|  | | | | | | | | |
|  | | | | | | | | |

|  | | | | | | |  | | |  | |
| --- | --- | --- | --- | --- | --- | --- | --- | --- | --- | --- | --- |
|  | | | | | | |  |  |  |  | |
|  | | | | | | | | | | | |
|  | 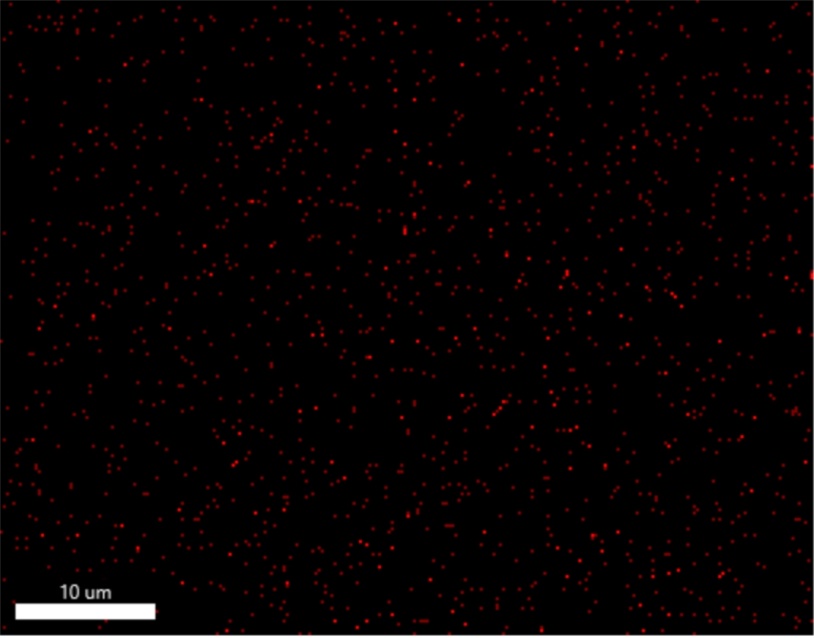 | |  | CuK_ROI (4) | | | |  | | | |
|  |  |  |  | | | | | | | | |
|  |  |  |  | | 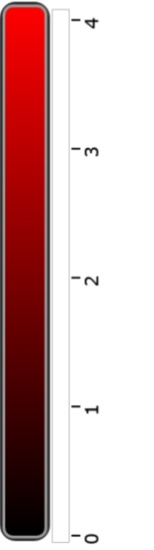 |  | | | | | |
|  |  |  |  | |  |  | | | | | |
|  |  |  |  | |  |  | | | | | |
|  | | | | | | | | | | | |
|  | 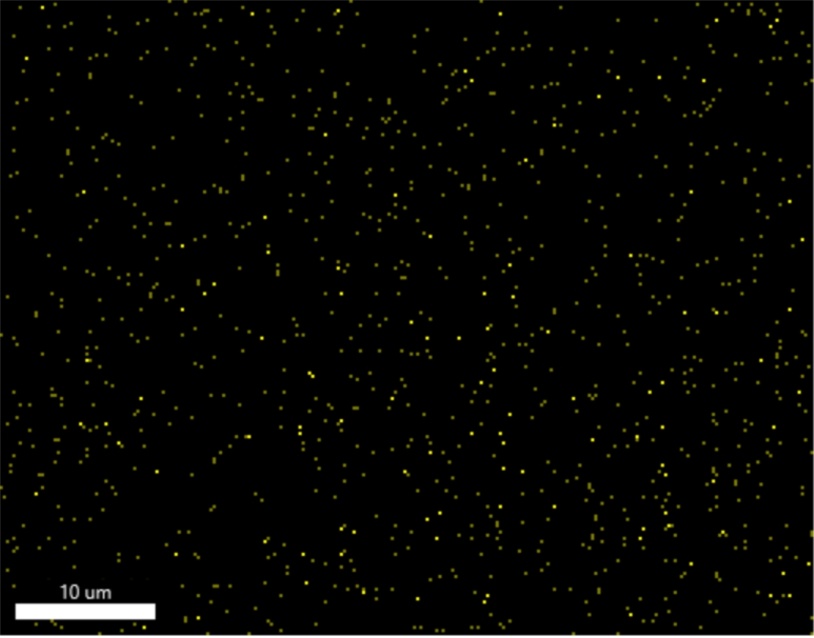 | |  | AsK_ROI (4) | | | |  | | | |
|  |  |  |  | | | | | | | | |
|  |  |  |  | | 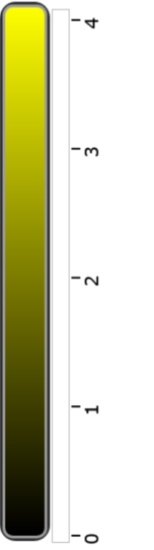 |  | | | | | |
|  |  |  |  | |  |  | | | | | |
|  |  |  |  | |  |  | | | | | |
|  | | | | | | | | | | | |
|  | | | | | | | | | | | |
|  | |  | | | | | | |  | |  |

**S. Fig. 5 (D)**

|  | | | | | | | | | | | | | |  | | | |  | | |
| --- | --- | --- | --- | --- | --- | --- | --- | --- | --- | --- | --- | --- | --- | --- | --- | --- | --- | --- | --- | --- |
|  | | | | | | | | | | | | | |  |  |  |  |  | | |
|  | | | | | | | | | | | | | | | | | | | |  |
|  | | | | | | | | | | | | | | | | | | | |  |
| kV: | 20 | Mag: | 2200 | Takeoff: | | 32.6 | |  | Live Time(s): | | 163.7 | Amp Time(µs): | | | | 3.84 | Resolution:(eV) | | 130 |  |
|  | | | | | | | **Sum Spectrum** | | | | | | |  | | | | | |  |
| 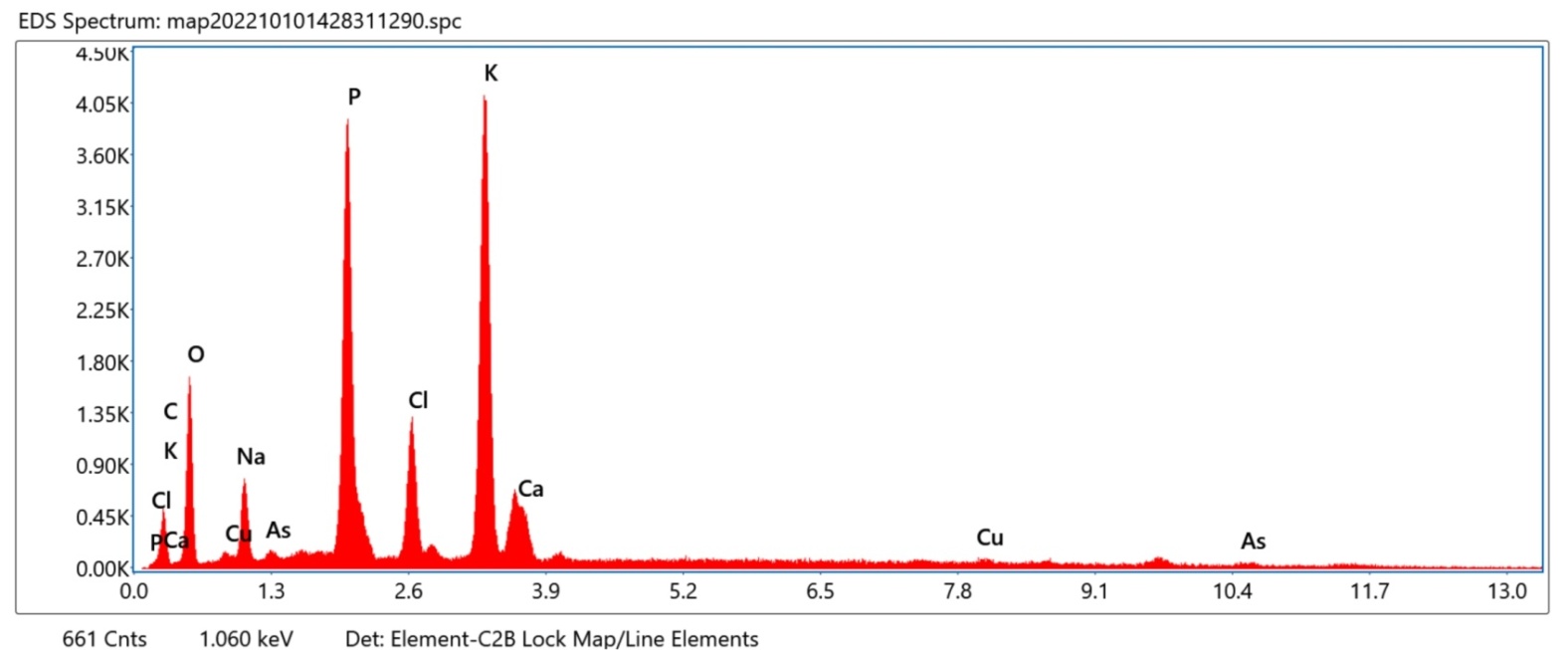 | | | | | | | | | | | | | | | | | | | |  |
|  |  |  |  |  |  |  |  |  |  |  |  |  |  |  |  |  |  |  |  |  |
|  |  |  |  |  |  |  |  |  |  |  |  |  |  |  |  |  |  |  |  |  |
|  |  |  |  |  |  |  |  |  |  |  |  |  |  |  |  |  |  |  |  |  |
|  | | | | | | | | | | | | | | | | | | | | |
| **Smart Quant Results** | | | | | | | | | | | | | | | | | | | |  |
|  | | | | | Element | | | | Weight % | Atomic % | | Error % |  | | | | | | | |
|  | | | | | C K | | | | 6.7 | 13.1 | | 15.7 |  | |  | | | | | |
|  | | | | | O K | | | | 30.9 | 45.2 | | 10.8 |  | |  | | | | | |
|  | | | | | NaK | | | | 5.8 | 5.9 | | 9.8 |  | |  | | | | | |
|  | | | | | P K | | | | 17.1 | 12.9 | | 3.7 |  | |  | | | | | |
|  | | | | | ClK | | | | 6.1 | 4.0 | | 4.3 |  | |  | | | | | |
|  | | | | |  |  |  |  |  |  |  |  |  |  |  | | | | | |
|  | | | | | K K | | | | 25.5 | 15.3 | | 2.9 |  | |  | | | | | |
|  | | | | | CaK | | | | 3.6 | 2.1 | | 6.9 |  | |  | | | | | |
|  | | | | | CuK | | | | 1.3 | 0.5 | | 15.5 |  | |  | | | | | |
|  | | | | | AsK | | | | 2.9 | 0.9 | | 16.3 |  | |  | | | | | |
|  | | | | |  |  |  |  |  |  |  |  |  |  |  | | | | | |
|  | | | | |  | | | |  |  | |  |  | |  | | | | | |

**Supplementary Table 1:-** NCBI Blast Data of ten closest neighbors of Sp2b

| **S.No.** | **Name of Organism** | **Accession No.** | **% Identity** |
| --- | --- | --- | --- |
| 1. | *Acinetobacter* sp. strain Sp2(b) 04 16S ribosomal RNA gene, partial sequence | OP010048.1 | 100% |
| 2. | *Acinetobacter calcoaceticus* strain BL5 16S ribosomal RNA gene, partial sequence | JQ923443.1 | 99.65% |
| 3. | *Acinetobacter* sp. strain WWTP-I-0520-A-V-5a 16S ribosomal RNA gene, partial sequence | MZ197948.1 | 99.54% |
| 4. | *Acinetobacter pittii* strain D1-316S ribosomal RNA gene, partial sequence | OR574182.1 | 99.42% |
| 5. | *Acinetobacter* sp. SFA2.516S ribosomal RNA gene, partial sequence | KU738963.1 | 99.42% |
| 6. | *Acinetobacter pittii* strain B35 16S ribosomal RNA gene, partial sequence | OP217083.1 | 99.42% |
| 7. | *Acinetobacter* sp. M.pstv .24.1 16S ribosomal RNA gene, partial sequence | KM108510.1 | 99.42% |
| 8. | *Acinetobacter pittii* strain CISH L116S ribosomal RNA gene, partial sequence | MZ268245.1 | 99.42% |
| 9. | *Acinetobacter pittii* DSM 2165316S ribosomal RNA gene, partial sequence | MN307289.1 | 99.42% |
| 10. | *Acinetobacter calcoaceticus* strain Xuyi 350 1 16S ribosomal RNA gene, partial sequence | MN240423.1 | 99.42% |
